# Supplementary material for: Stability criteria for the consumption and exchange of essential resources
Source: PLoS Comput Biol. 2022 Sep 8;18(9):e1010521. doi: 10.1371/journal.pcbi.1010521 (PMC9488833; doi:10.1371/journal.pcbi.1010521)
Supplement: S1 Appendix — We provide proofs of the main results, additional numerical simulations and descriptions of all of the matrix parameterizations. (PDF) [file pcbi.1010521.s001.pdf]

S1 Appendix: Stability Criteria for the Consumption and Exchange of Essential Resources

Theo Gibbs [1], Yifan Zhang [2], Zachary R. Miller [3], James P. O'Dwyer [2]

[1] Lewis-Sigler Institute for Integrative Genomics, Princeton University, Princeton, New Jersey, USA

[2] Department of Plant Biology, University of Illinois, Urbana, Illinois, USA

[3] Department of Ecology & Evolution, University of Chicago, Chicago Illinois, USA

Contents

1 Model Form and Simplifications **3**

2 Stability Criteria **3**

2.1 Deriving the Jacobian 3

2.2 Sufficient conditions for stability 4

2.3 Gershgorin bound implies that B is negative definite 7

2.4 Stability criteria for the example consumption and production patterns 7

2.5 Stability criteria without cross-feeding 8

2.6 Calculating the eigenvalues of J when A and B are symmetric and simultaneously diagonalizable 9

2.7 Is J stable if and only if B is stable when B is symmetric? 10

2.8 Intuition for why small consumer abundances cause instability 12

3 Feasibility Analysis **12**

3.1 General feasibility criteria 12
3.2 Feasibility for the example consumption and production patterns 13
3.3 Sufficient stability criteria imply feasibility 13
3.4 Feasibility for different growth rules 14
3.5 Variable resource abundances 14

4 Matrix Parameterizations **15**

4.1 Consumption matrices 15
4.2 Production matrices 16

1 Model Form and Simplifications

We consider the consumer-resource model

$$\begin{aligned}
\dot{R}_i &= \rho_i - R_i \sum\nolimits_j C_{ij} N_j + \sum\nolimits_j P_{ji} \sum\nolimits_k \left(C_{jk} R_j - \frac{g_k}{\epsilon_{jk}} \right) N_k + \theta \sum\nolimits_j \tilde{P}_{ji} \sum\nolimits_k \frac{g_k}{\epsilon_{jk}} N_k \\
\dot{N}_k &= N_k \left((1-\theta) g_k - \eta_k \right) = N_k \left((1-\theta) \min_{j \in \{1,\dots,S\}} \{ \epsilon_{jk} C_{jk} R_j : C_{jk} \neq 0 \} - \eta_k \right)
\end{aligned} \tag{S1}$$

with parameters as defined in the main text. Throughout our simulations and analytical theory, we generally set the ϵ_{ii} values all to some small fraction and set the remaining $\epsilon_{ij} = 1$ for $i \neq j$, meaning that the diagonal consumer-resource pairs are limiting. We focus on this case mainly for convenience, as it simplifies our numerical and mathematical analysis.

In addition, there can be added complexity in this model when consumers do not require and therefore deplete all of the resources in the system. In this case, the sum over the $\tilde{P}_{ji}$ parameters will not actually be over all of the k indices, because only those resources that are actually consumed will lead to cross-feeding. Similarly, consumer growth rates are actually dependent on the total number of resources that each consumer depletes. When consumers don't deplete all resources, this number can vary between consumers. Throughout our analysis, we measure consumer abundances in units that eliminate this additional multiplicative factor in consumer growth rates, simplifying the model.

2 Stability Criteria

In this section, we derive the sufficient stability criteria described in the main text. We show that, if a fixed point exists in the model dynamics and if our stability criteria are satisfied, the fixed point is guaranteed to be stable. We describe how these stability criteria apply to our example consumption and production structures. Last, we discuss under what conditions our sufficient stability criteria are also necessary.

2.1 Deriving the Jacobian

Suppose that the model in Eq. (1) of the main text admits a fixed point with (positive) abundances $\vec{R}^\star$ and $\vec{N}^\star$. As discussed in the main text, near to a fixed point, the model reduces to the dynamical

system in Eq. (2), so we evaluate the Jacobian $\mathbf{J}$ of Eq. (2) at the fixed point $\vec{R}^\star$ and $\vec{N}^\star$ to determine its stability. If the eigenvalues of the Jacobian all have negative real part, then the equilibrium is stable to small perturbations. Let's compute the Jacobian of this system:

$$\begin{aligned}
\frac{\partial \dot{R}_i}{\partial R_j} &= -\delta_{ij} \sum\nolimits_k C_{ik} N_k + P_{ji} \sum\nolimits_k C_{jk} N_k - C_{jj} N_j \sum\nolimits_k P_{ki} \tilde{\epsilon}_{kj} + \theta\, C_{jj} N_j \sum\nolimits_k \tilde{P}_{ki} \tilde{\epsilon}_{kj} \\
\frac{\partial \dot{R}_i}{\partial N_j} &= -R_i C_{ij} + \sum\nolimits_k P_{ki} C_{kj} R_k - C_{jj} R_j \sum\nolimits_k P_{ki} \tilde{\epsilon}_{kj} + \theta\, C_{jj} R_j \sum\nolimits_k \tilde{P}_{ki} \tilde{\epsilon}_{kj} \\
\frac{\partial \dot{N}_i}{\partial R_j} &= \delta_{ij} (1 - \theta)\, N_i \epsilon_{ii} C_{ii} \\
\frac{\partial \dot{N}_i}{\partial N_j} &= 0
\end{aligned} \tag{S2}$$

In matrix notation, the Jacobian is

$$J = \begin{bmatrix} -[C\vec{N}]_d + P^T [C\vec{N}]_d - P^T \tilde{\epsilon} C_d N_d + \theta \tilde{P}^T \tilde{\epsilon} C_d N_d & -\vec{R}_d C + P^T \vec{R}_d C - P^T \tilde{\epsilon} C_d \vec{R}_d + \theta \tilde{P}^T \tilde{\epsilon} C_d \vec{R}_d \\ (1-\theta) \vec{N}_d \epsilon_{diag} C_{diag} & 0 \end{bmatrix} \tag{S3}$$

where C_{diag} (and similarly ϵ_{diag}) is the diagonal matrix with entries from the diagonal of C (respectively, ϵ). For convenience in the calculations ahead, we will define the short-hand notations A and B for the upper-left and upper-right blocks of J, respectively (so we have $A = -[C\vec{N}]_d + P^T [C\vec{N}]_d - P^T \tilde{\epsilon} C_d N_d + \theta \tilde{P}^T \tilde{\epsilon} C_d N_d$ and $B = -\vec{R}_d C + P^T \vec{R}_d C - P^T \tilde{\epsilon} C_d R_d + \theta \tilde{P}^T \tilde{\epsilon} C_d R_d$). Note that we abuse notation here by including the abundances $\vec{R}_d$ in this definition of B, which is slightly different from the definition in the main text. However, in the cases we focus on analytically, this new B is directly proportional to the B in the main text. In the following sections, we will derive criteria for the stability of the Jacobian under some additional assumptions.

2.2 Sufficient conditions for stability

First, we show that 0 is not an eigenvalue of J, so that we can invert the matrix $-\lambda I$ (where λ is an eigenvalue of J) when applying block determinant rules. Suppose 0 is an eigenvalue of J; then J is singular and there exists a non-zero vector, with first S components denoted by $\vec{v}$, and second S components denoted by $\vec{w}$, such that

$$J \begin{bmatrix} \vec{v} \\ \vec{w} \end{bmatrix} = \begin{bmatrix} A & B \\ (1-\theta) \vec{N}_d \epsilon_{diag} C_{diag} & 0 \end{bmatrix} \begin{bmatrix} \vec{v} \\ \vec{w} \end{bmatrix} = \vec{0}\,. \tag{S4}$$

Since $(1-\theta)\vec{N}_d \epsilon_{diag} C_{diag}$ is a diagonal matrix with positive entries by assumption, it is invertible and we know that $\vec{v}=\vec{0}$. Therefore, $B\vec{w}=\vec{0}$. But we notice that $-B^{-1}$ is precisely the matrix that defines $\vec{N}^\star$ in Eq. (3) of the main text. So B is also invertible by our assumption that $\vec{N}^\star$ is well-defined, and we know that $\vec{w}=0$. This contradicts our initial assumption that $(\vec{v},\vec{w})$ is non-zero. Thus, we conclude that 0 is not an eigenvalue of J.

Now, we apply block determinant rules to the matrix $J-\lambda I$ and compute the characteristic polynomial for J:

$$\det[J-\lambda I] = \det[-\lambda I]\det\left[A-\lambda I+\frac{1-\theta}{\lambda}B\vec{N}_d\epsilon_{diag}C_{diag}\right] \tag{S5}$$

As we have ruled out $\lambda=0$, this implies that the eigenvalues of J are defined by

$$\det\left[A-\lambda I+\frac{1-\theta}{\lambda}B\vec{N}_d\epsilon_{diag}C_{diag}\right]=0 \tag{S6}$$

At this point we concentrate on a simplified version of the model, in which $\vec{R}^\star = r\vec{1}$ and $\vec{N}^\star = n\vec{1}$, where $\vec{1}$ is a vector of all ones. We also assume that $C_{ii}=C_d$ and $\epsilon_{ii}=\epsilon_d$ for all i. Now the relevant matrices in the characteristic polynomial simplify to $A = n\left(-[C\vec{1}]_d + P^T[C\vec{1}]_d - C_d P^T\tilde{\epsilon} + \theta C_d \tilde{P}^T\tilde{\epsilon}\right)$ and $B' = \frac{1-\theta}{\lambda}B\vec{N}_d\epsilon_{diag}C_{diag} = \frac{1}{\lambda}(1-\theta)nr\epsilon_d C_d\left(-C+P^TC - C_d P^T\tilde{\epsilon}+\theta C_d\tilde{P}^T\tilde{\epsilon}\right)$.

We aim to show that, given the two stability criteria described in the main text, the eigenvalues of J must have strictly negative real parts. We use proof by contradiction: Assume that there is an eigenvalue λ with Re$(\lambda)>0$. Then we will demonstrate that the matrix $A+B'$ has all eigenvalues with strictly negative real part. Eq. (S5) shows that λ are also the eigenvalues of $A+B'$, so this results in a contradiction. Therefore, we will conclude that Re$(\lambda)<0$.

First, notice that the stability criteria directly imply that B, and hence B', given the assumption Re$(\lambda)>0$, is negative definite. Next, observe that A can be expressed as

$$A = n\left(-[C\vec{1}]_d + P^T[C\vec{1}]_d + C - P^TC + \frac{1}{r}B\right) = n\left(-(I-P^T)([C\vec{1}]_d - C) + \frac{1}{r}B\right) \tag{S7}$$

The positive factor n will not affect our conclusions, so we neglect it for simplicity. By the stability criteria in the main text, the matrices $I-P^T$ and $[C\vec{1}]_d - C$ are individually symmetric. In fact, these matrices are (weakly) diagonally dominant, and so the Gershgorin circle theorem implies that they are individually positive semi-definite [1]. In particular, each matrix has an eigenvector $\vec{1}$ with corresponding eigenvalue 0; then assuming P and C are invertible, the remaining $S-1$ eigenvalues of the above matrices are strictly positive. For these conclusions, we use the fact that P is column stochastic (i.e. $P^T\vec{1}=\vec{1}$). However, the product $(I-P^T)([C\vec{1}]_d - C)$ is not generally

symmetric, and so not positive semi-definite.

We will now show that despite this non-symmetry, A shares the same eigenvalues as a negative definite matrix, and thus has all real negative eigenvalues. Before proceeding, it is necessary to deal with a small technical detail. We have seen that $[C\vec{1}]_d - C$ has one zero eigenvalue, which renders this matrix uninvertible. This will complicate our calculations, so we are motivated to express A as

$$\begin{aligned} A &= -(I - P^T)([C\vec{1}]_d - C) + c\,(I - P^T) + c\,(I - P^T) + \frac{1}{r}B \\ &= -(I - P^T)([C\vec{1}]_d - C - cI) + \left(c\,(I - P^T) + \frac{1}{r}B\right) \end{aligned} \tag{S8}$$

where c is an arbitrary small constant. For c sufficiently small, the matrix $c\,(I - P^T) + \frac{1}{r}B = B''$ is negative definite, because B is negative definite and the eigenvalues are a continuous function of the matrix entries (this can be shown formally using Weyl's inequality). But now $[C\vec{1}]_d - C - cI$ is negative definite, and in particular invertible. Let $V\Lambda V^T$ be the eigendecomposition of $[C\vec{1}]_d - C - cI$. The matrix $Q = \Lambda^{1/2}V^{-1}(A + B')V\Lambda^{-1/2} = \Lambda^{1/2}V^T(A + B')V\Lambda^{-1/2}$ is similar to $A + B'$, and thus shares the same eigenvalues. Expanding Q, we have

$$\begin{aligned} Q &= -\Lambda^{1/2}V^T(I - P^T)([C\vec{1}]_d - C - cI)V\Lambda^{-1/2} + \Lambda^{1/2}V^T B''V\Lambda^{-1/2} + \Lambda^{1/2}V^T B'V\Lambda^{-1/2} \\ &= -\Lambda^{1/2}V^T(I - P^T)V\Lambda^{1/2} + \Lambda^{1/2}V^T B''V\Lambda^{-1/2} + \Lambda^{1/2}V^T B'V\Lambda^{-1/2} \end{aligned} \tag{S9}$$

The first term of Q, in the second line above is *congruent* to $-(I - P^T)$, which is a negative semi-definite matrix. Then Sylvester's law of inertia implies that this term is also negative semi-definite [1]. The remaining two terms are similar to negative definite matrices (B'' and B', respectively), implying that they are also negative definite. Thus, Q is a sum of negative definite and negative semi-definite matrices. This implies that Q is negative definite, and therefore Q and $A + B'$ have all real negative eigenvalues. This contradicts the assumption that Re$(\lambda) > 0$, so we have proved that the criteria in the main text are sufficient to ensure the stability of J.

2.3 Gershgorin bound implies that B is negative definite

A straightforward application of the Gershgorin circle theorem to the matrix B yields a sufficient condition for the stability of B that provides additional ecological insight. If $H(B)_{ii}+\sum_{j\neq i}|H(B)_{ij}| < 0$ for each i, the eigenvalues of the Hermitian part of B are all contained in the left half of the complex plane and $H\left(\frac{1}{\lambda}B\right)$ is negative definite. These inequalities demonstrate that, if B has large diagonal coefficients, then the system is more likely to be stable. Because the diagonal coefficients of B are proportional to the diagonal consumption coefficients, this relationship helps to explain why C_d is a key bifurcation parameter in the model. In Fig D and E, we plot the predictions for this sufficient Gershgorin condition in addition to the predictions from the second stability condition we stated in the main text.

2.4 Stability criteria for the example consumption and production patterns

We now show that the example consumption and production structures which we described in the main text automatically generate symmetric B matrices, satisfying our first sufficient stability criterion. We consider the two terms of the matrix

$$B = r\left[\left(I - P^T\right)C + \left(P^T - \theta\tilde{P}^T\right)\tilde{\epsilon}\vec{C}_d\right] \tag{S10}$$

separately.

In the tradeoff parameterization, $P_{ij} = \frac{1}{S-1}$ when $i \neq j$ while $P_{ii} = 0$. C is a symmetric matrix with row (or column) sums $\sum_j C_{ij} = C_d + (S-1)C_0$ for each i. The (i,j)-th entry of the matrix product P^TC is $[P^TC]_{ij} = \frac{1}{S-1}\left(C_d + (S-1)C_0 - C_{ij}\right)$, while the (j,i)-th entry is $[P^TC]_{ji} = \frac{1}{S-1}\left(C_d + (S-1)C_0 - C_{ji}\right)$. Since C is symmetric, $C_{ij} = C_{ji}$ and P^TC is symmetric as well. Therefore, B is symmetric since it is the sum of symmetric matrices, and the first stability condition is satisfied. Circulant matrices commute with one another, and $\left(P^T - \theta\tilde{P}^T\right)\tilde{\epsilon}\vec{C}_d = C_d\left(P^T - \theta\tilde{P}^T\right)\tilde{\epsilon}$ is the product of two symmetric circulant matrices, and therefore is symmetric itself. Overall, B is symmetric.

Now, we consider the symmetric circulant matrix case. Since C and P^T are both symmetric and circulant, we have that $(P^TC)^T = C^TP = CP^T = P^TC$. In other words P^TC is symmetric. Similarly, the second term in the B matrix is also a product of two symmetric circulant matrices. Therefore, B is symmetric and the first condition is always satisfied. It is possible for the second condition to be false, so we need to explicitly check this condition, as we do in the main text.

2.5 Stability criteria without cross-feeding

When there is no cross-feeding, $P_{ij} = \tilde{P}_{ij} = 0$ for all i, j. In this section, we assume that $\sum_{j \neq i} C_{ij} = (S-1)C_0$ for analytical tractability, and we also continue to assume that $\vec{R}^\star = r\vec{1}$, $\vec{N}^\star = n\vec{1}$, $C_{ii} = C_d$ and $\epsilon_{ii} = \epsilon$ for all i. The eigenvalues λ of the Jacobian J satisfy the characteristic polynomial

$$0 = \det(J - \lambda I) = \det\left(\begin{bmatrix} -[C\vec{N}]_d - \lambda I & -\vec{R}_d C \\ \vec{N}_d \epsilon_d C_{diag} & -\lambda I \end{bmatrix}\right) = \det\left(\begin{bmatrix} -n((S-1)C_0 + C_d)I - \lambda I & -rC \\ nC_d I & -\lambda I \end{bmatrix}\right). \tag{S11}$$

First, let's decide if $\lambda = -n((S-1)C_0 + C_d)$ can be an eigenvalue. Let $v, w \in \mathbb{R}^S$ and suppose that

$$\begin{bmatrix} 0 & -rC \\ n\epsilon C_d I & n((S-1)C_0 + C_d)I \end{bmatrix} \begin{bmatrix} v \\ w \end{bmatrix} = \vec{0}\,. \tag{S12}$$

Since $-rC$ is invertible, $w = \vec{0}$, but then $v = \vec{0}$ as well, and $\lambda = -n((S-1)C_0 + C_d)$ is not an eigenvalue of J. Therefore, we can safely apply block determinant rules to find all the eigenvalues. We find that

$$0 = \det\left(-\vec{N}_d \epsilon_d C_{diag}\left([C\vec{N}]_d + \lambda I\right)^{-1} \vec{R}_d C - \lambda I\right) = \det\left(-\frac{nr\epsilon C_d}{n((S-1)C_0 + C_d) + \lambda} C - \lambda I\right) \tag{S13}$$

so we can write down the eigenvalues of J in terms of the eigenvalues of C. If ω is an eigenvalue of C, then the roots of the polynomial

$$\lambda^2 + n((S-1)C_0 + C_d)\lambda + nr\epsilon C_d \omega = 0 \tag{S14}$$

are eigenvalues of J. So, we want to know when the roots of this complex quadratic have negative real part. We can derive constraints on ω under which λ is guaranteed to have negative real part by using the quadratic formula and the identity $Re\left(\sqrt{x+iy}\right) = \frac{1}{\sqrt{2}}\sqrt{\sqrt{x^2+y^2} + x}$, or just by appealing to the Routh-Hurwitz stability criteria. In either case, if

$$\begin{aligned} &n((S-1)C_0 + C_d) > 0 \\ &n((S-1)C_0 + C_d)^2 Re(\omega) > rC_d Im(\omega)^2 \end{aligned} \tag{S15}$$

hold, then $Re(\lambda) < 0$. The first constraint in (S15) is always satisfied from the definitions. The second constraint is the interesting one. First of all, if $Re(\omega) \leq 0$ (ie. if $-C$ is unstable), then the Jacobian J is unstable, since the right hand side of (S15) is non-negative. So, we can already rule out consumption structures (C matrices) that are unstable by themselves. In addition to the stability of C, (S15) shows that the magnitude of $Im(\omega)$ must be small compared the magnitude of $Re(\omega)$. If $Im(\omega) \neq 0$, which is true for a generic consumption matrix, then by sending $n \to 0$, we will eventually get instability. These results directly mirror those presented in the main text, where

low consumer abundances lead to instability. Moreover, the criteria we have derived in this simpler case unify our two stability criteria in the more general case. To see this, let's interpret our more general stability criteria in the non-cross-feeding parameterization. The first symmetry criterion in the general case forces the imaginary part of the eigenvalues of C to be zero ($Im(\omega) = 0$) and the second stability criterion ensures that the matrix C is itself stable. In the non-cross-feeding case, however, we see that these stability criteria are overly restrictive. It is possible to have stability without necessarily being precisely symmetric (and therefore forcing $Im(\omega) = 0$), as long as the real parts of the eigenvalues of the matrix C are large enough. These results mirror our conjecture in the main text that if the matrix B has small imaginary parts, then it will be protected from stability at low consumer abundances (see also Fig I). In addition, the bound in (S15) predicts that, for larger values of r, the system will become unstable at larger values of n, which we then observe for our more complex model in Fig F.

2.6 Calculating the eigenvalues of J when A and B are symmetric and simultaneously diagonalizable

In the previous section, we showed how the eigenvalues of J are directly related to the eigenvalues of C when there is no cross-feeding. Now, we prove a similar result in a different context. We show that the eigenvalues of J can be written in terms of the eigenvalues of the matrices A and B provided that these matrices are simultaneously diagonalizable. In particular, the two consumption and production structures which we present in the main text (the tradeoff and circulant cases) give rise to A and B matrices which are simultaneously diagonalizable.

In the proof of our sufficient stability criteria, we showed that each non-zero eigenvalue λ of J satisfies

$$0 = \det\left[\lambda A + n\epsilon r C_d B - \lambda^2 I\right] \tag{S16}$$

which means that the matrix $\lambda A + n\epsilon r C_d B - \lambda^2 I$ has an eigenvalue of 0. Equivalently, there exists an vector $\vec{v}$ such that

$$\lambda A\vec{v} + n\epsilon r C_d B\vec{v} = \lambda^2 \vec{v} \tag{S17}$$

for each eigenvalue λ of J. When A and B are simultaneously diagonalizable, they share the same eigenspaces, so we can find a vector $\vec{v}$ that is an eigenvector of both A and B. Let λ_A denote the corresponding eigenvalue of A and λ_B the corresponding eigenvalue of B. Then, we can re-write the above vector equation in terms of these eigenvalues and solve for λ to get that

$$\lambda = \frac{1}{2}\left(\lambda_A \pm \sqrt{\lambda_A^2 + 4n\epsilon r C_d \lambda_B}\right) \tag{S18}$$

so we have solved for the eigenvalues of J in terms of the corresponding pairs of eigenvalues

of A and B. Using Eq. S18, we can also prove that J is stable if and only if B is stable when B is symmetric. If A and B are symmetric, then both λ_A are λ_B are real. In this case, $\sqrt{\lambda_A^2 + 4n\epsilon r C_d \lambda_B} \leq \lambda_A$ as long as $\lambda_B < 0$, so that $\lambda < 0$ and the equilibrium is stable. Since B is symmetric, we know that $\lambda_A < 0$, so the only way to have a positive λ is to have $\lambda_B > 0$. In other words, the sign of λ is simply given by the sign of λ_B and J is stable if and only if B is stable. In Fig J, we plot the predictions of Eq. S18 as well as the spectrum of J and the agreement is exact.

2.7 Is J stable if and only if B is stable when B is symmetric?

We have shown in two different simplified scenarios that J is stable if and only if B is stable provided that B is symmetric, which suggests that this may be true in general. We cannot prove this statement in its full generality. In this section, we first provide additional numerical evidence that it is true. We consider a more general parameterization of the Jacobian:

$$J = \begin{bmatrix} A & B \\ nI & 0 \end{bmatrix} \tag{S19}$$

where A is an arbitrary negative definite matrix, B is an arbitrary symmetric matrix and n is some positive constant. Note that these new definitions of A, B and n slightly abuse the notation we have developed because they absorb additional constants (such as ϵ and r), but this choice does not affect any of the qualitative results. This more general parameterization of J includes the Jacobian we have analyzed, but it removes the relationship between A and B that exists in our model. Instead, A and B are arbitrary matrices, as long as they are negative definite and symmetric respectively. In Fig K, we plot the maximum eigenvalue of B against the real part of the eigenvalue of J with largest real part for A and B matrices with randomly sampled entries and for a few different system sizes S. We vary the diagonal entries of B to create stable and unstable matrices (see the R code on Github at https://github.com/theogibbs/essential-stability-criteria for details). Across many replicates, the sign of the leading eigenvalue of B predicts exactly the sign of the leading eigenvalue of J even in this more general parameterization of the Jacobian.

We have already proven that, if B is stable, then J is stable when B is symmetric. One way to prove the reverse implication is to show that, if B is unstable, then so is J. We will actually show that, if B is unstable with an odd number of positive eigenvalues and no repeated eigenvalues, then J is unstable. Since B has no repeated eigenvalues, it admits an eigendecomposition. Therefore, there exists a matrix Q such that $B = Q^{-1}\Lambda Q$ where Λ is a diagonal matrix containing the eigenvalues of B. Because B is symmetric, we also know that Q is orthogonal so that $Q^{-1} = Q^T$. Now, let's set

$$U = \begin{bmatrix} Q & 0 \\ 0 & Q \end{bmatrix} \tag{S20}$$

so that we can define the matrix

$$J_1 = U^{-1}JU = \begin{bmatrix} Q^{-1}AQ & \Lambda \\ nI & 0 \end{bmatrix} . \tag{S21}$$

J_1 is similar to J, so they share the same eigenvalues. Let's denote the eigenvalues of B by $\lambda_1 > \lambda_2 > \cdots > \lambda_k > 0 > \lambda_{k+1} > \cdots > \lambda_S$ for some odd integer $k \in \{1, \ldots, S\}$ and let's define the matrix $|\Lambda|$ as the entry-wise absolute value of the matrix Λ. Let's also define the matrix T as a $2S \times 2S$ block matrix with $S \times S$ size blocks. The off-diagonal blocks of T are all zeroes, the upper left-hand block is the identity and the lower right-hand block is a diagonal matrix with -1 in the first k columns and 1 in the remaining columns. Then, we can write the matrix J_1 as

$$J_1 = \begin{bmatrix} Q^{-1}AQ & -|\Lambda| \\ nI & 0 \end{bmatrix} T = J_2 T \tag{S22}$$

where we have defined J_2. Last, let's define

$$J_3 = UJ_2U^{-1} = \begin{bmatrix} A & Q\left(-|\Lambda|\right)Q^{-1} \\ nI & 0 \end{bmatrix} . \tag{S23}$$

We have already shown that J_3 is stable because it satisfies our two stability criteria. In particular, $Q|\Lambda|Q^{-1}$ is symmetric because Q is orthogonal: $[Q\left(-|\Lambda|\right)Q^{-1}]^T = Q^{-T}\left(-|\Lambda|\right)Q^T = Q\left(-|\Lambda|\right)Q^{-1}$. It is also similar to the matrix $-|\Lambda|$ so it has negative eigenvalues and is negative definite. Similarly, A is negative definite by assumption. Therefore, J_3 is stable and so is J_2 because they are similar. On the other hand, $\det[J_2] = -\det[J_1]$ because $\det[T] = -1$ since k is odd. From this, we can conclude that J has at least one positive real eigenvalue. Since J is a real matrix, its complex eigenvalues come in complex conjugate pairs. The determinant is the product of the eigenvalues, so these conjugate pairs do not change its sign, regardless of whether or not they have positive or negative real part, since they enter the determinant only through their modulus. Since J_1 and J_2 have opposite signs, we know that at least one of the real eigenvalues of J_1 must be positive. If this were not true, then J_1 and J_2 would have determinants with the same sign. So, because J is similar to J_1, J is unstable as well.

Although we have not shown the if-and-only-if statement in full generality, we have shown that the transition from stability to instability is sharp when B does not have degenerate eigenvalues. Let's consider slowly changing some parameter (like C_d) such that B just becomes unstable. Then, B has only one positive eigenvalue because it does not have any degenerate eigenvalues. From the above argument, we know that J is also unstable. We cannot analytically rule out the possibility that J becomes stable after more of the eigenvalues of B become positive, though we do not observe this behavior in simulations.

2.8 Intuition for why small consumer abundances cause instability

As the consumer abundances are reduced in our model, the spectrum of the Jacobian begins to have many eigenvalues with non-zero imaginary parts. These complex eigenvalues are separated into two clouds above and below the real axis (see Fig M). The imaginary parts of the eigenvalues of B control the width of these clouds, while the consumer abundances determine where they are centered. If B has purely real eigenvalues, then these two clouds have no width, so regardless of how small the consumer abundances become, no eigenvalues will cross over the imaginary axis. If instead B has eigenvalues with non-zero imaginary part, then the clouds in the spectrum of J will have non-zero width, and some of these eigenvalues will cross the imaginary axis at a small value of n, creating an unstable fixed point. This description also helps to explain why the interaction network B need not be precisely symmetric to still promote, but not guarantee, stability. As the imaginary parts of the eigenvalues of B become smaller, so too does the width of the eigenvalue clouds in the spectrum of J. As a result, networks which are not precisely symmetric but do have eigenvalues with small imaginary parts are better protected from instability at low consumer abundance than those with larger imaginary parts.

3 Feasibility Analysis

Throughout the stability analysis in the previous section, we assumed that a fixed point $\vec{R}^\star$ and $\vec{N}^\star$ existed. We now evaluate when such a fixed point exists, and describe how our feasibility criteria relate to our stability criteria.

3.1 General feasibility criteria

As we stated in the main text, there is a fixed point in the simplified model (where consumers do not change their resource preferences as a function of resource availability) when the abundances

$$\vec{N}^\star = \left[\left(I - P^T\right)\vec{R}^\star_d C + \left(P^T - \theta\tilde{P}^T\right)\tilde{\epsilon}\vec{C}_d\vec{R}^\star_d\right]^{-1}\vec{\rho} \qquad \text{(S24)}$$

are all positive for the specified resource inflow $\vec{\rho}$. We can solve for the resource abundances immediately because the consumer dynamics involve only one resource. We get that $R^\star_i = \frac{\eta_i}{(1-\theta)\epsilon_{ii}C_{ii}}$.

3.2 Feasibility for the example consumption and production patterns

We now show that the abundances $\vec{N} = n\vec{1}$ is a fixed point for the example consumption and production structures that we consider in the main text. We need the vector

$$\vec{\rho} = nr\left[\left(I - P^T\right)C + \left(P^T - \theta\tilde{P}^T\right)\tilde{\epsilon}\vec{C}_d\right]\vec{1} \tag{S25}$$

to have all positive components. In each of our parameterizations, the C, P and $\tilde{P}$ matrices are symmetric and have constant row and column sums. Specifically, $\left(I - P^T\right)C\vec{1} = ((S-1)C_0 + C_d)\left(I - P^T\right)\vec{1} = \vec{0}$, while $\left(P^T - \theta\tilde{P}^T\right)\tilde{\epsilon}\vec{C}_d\vec{1} = (1-\theta)C_d[\tilde{\epsilon}\vec{1}]$ which has all positive components. Therefore, this constant supply vector ($\vec{\rho} = (1-\theta)C_d[\tilde{\epsilon}\vec{1}]$) is always positive by definition, so the example consumption and production structures are always feasible. For more complex resource inflows, however, $\vec{N}$ is not a constant vector and we have not proven that these interaction structures guarantee feasibility. Moreover, the unstructured network structures can give rise to feasible or unfeasible abundances, even when the abundances are constant (see Fig E).

3.3 Sufficient stability criteria imply feasibility

The Gershgorin bound that implies the second stability criterion requires the row sums of the matrix $\bar{B}$ to be negative, where $\bar{B}$ is the same as the matrix B except with the absolute value of the off-diagonal entries rather than their original values. For feasibility in the case of constant abundances, we need the row sums of the matrix $-B$ to be positive. Therefore, the Gershgorin bound that implies the second stability criterion is a more restrictive condition than feasibility in this case. In Fig E, we can see the affect of feasibility on the different matrix parameterizations as we vary the parameter ϵ. In the tradeoff and circulant cases, we proved in the previous section that these matrices are always feasible. As a result, the points which are the numerically determined stability thresholds do not change as ϵ changes, while the Gershgorin bound (the dashed lines in the figure) does change, because it is sensitive to changes in ϵ. For the unstructured case, both the numerically computed thresholds and the Gershgorin bounds change as a function of ϵ. This is because we enforce both feasibility and stability when we compute the numerical thresholds in Fig E, so the analytical bound is capturing the behavior of the probability of feasibility at low ϵ, rather than the probability of stability. By contrast, in Fig D, we only enforce stability in the numerically determined C_d values, so the difference between Fig D and Fig E reveals the influence of feasibility on the system.

3.4 Feasibility for different growth rules

Thus far, we have only discussed feasibility in the context of the simplified model where consumers do not change their preferred resource. However, given a feasible fixed point in this simpler model, it is not necessarily achieved in the dynamics of the model with a specified growth rule. For example, there could be a feasible fixed point in which a given consumer actually derives less benefit from a different resource than the one it is consuming. Under Liebig's law, the consumer switches to this resource, meaning that the feasible fixed point for the simplified model is not an equilibrium of the Liebig model. Under Liebig's law, we need

$$\min_{j \in \{1,\ldots,S\}} \{\epsilon_{jk} C_{jk} R_j : C_{jk} \neq 0\} = \epsilon_{ii} C_d \tag{S26}$$

for the assignment of consumer to resources to generate the feasible fixed point. In this equation, the ϵ matrix, in combination with the consumption coefficients and the resource abundances, determines which resources are limiting for each species. Therefore, if the the diagonal efficiencies ϵ_{ii} are small relative to the off-diagonal efficiencies, then the diagonal resources are limiting for the Liebig law growth rule. In Fig 5 of the main text and in Fig G, we fix ϵ and vary C_d which means that the minimum above is eventually realized by a different assignment of consumers to resources. Liebig's law introduces an upper limit on feasible C_d values. For a growth rule with a maximum instead of a minimum, the dependence is reversed. In this case, because C_d is large compared with the remaining consumption coefficients in order to ensure stability, the maximum is automatically realized by the diagonal consumer-resource pairs in the growth dynamics, and the ϵ matrix does not need to have a small diagonal.

3.5 Variable resource abundances

In Fig 5 of the main text, we showed that our qualitative stability conclusions still applied to a model where resource inflows were not chosen to ensure that all consumers had the same abundance. We still set $\eta_i = \eta$, however, so all resource abundances were equal (as in a chemostat with a constant dilution rate). In Fig H, we plot the analogous probabilities as in G, except we now sample the washout rates η_i from a uniform distribution. As a result, the resource abundances now vary between resources, but we observe the same qualitative transition into instability at low resource supply rates.

4 Matrix Parameterizations

In this section, we summarize the different matrix parameterizations we used to generate Fig G which test our hypothesis that the imaginary parts of the eigenvalues of B control stability. All the code used to run simulations and generate the figures is available on GitHub at https://github.com/theogibbs/essential-stability-criteria. In each case, we set the diagonal values of the consumption coefficients to be C_d. We also set the diagonal values of the P matrix to be zero. In Fig B, we visualize all of the consumption and production matrices we did not display in the main text.

4.1 Consumption matrices

Tradeoff: C is a symmetric matrix with row (or column) sums given by $\sum_j C_{ij} = C_d + (S-1)C_0$ for each i. To produce this parameterization numerically, we generate a random symmetric matrix from the underlying uniform distribution. Then, we find the closest matrix with constant row sums using the R package Spbsampling [2].

Circulant: First, we sample a random vector of length S from the underlying uniform distribution. Then, we assign the matrix elements in the permuted fashion described in the main text. Last, we make the matrix symmetric by taking its Hermitian part.

Unstructured: We randomly sample all the off-diagonal entries from the underlying uniform distribution.

Banded: We randomly sample the off-diagonal entries displaced from the diagonal by one index from the underlying uniform distribution. Then, we constrain each row to have a row sum of C_0. Therefore, the matrix B when P is constant has only real eigenvalues but is not symmetric.

Correlated: We specify a parameter p which controls the degree of correlation between each off-diagonal pair (C_{ij}, C_{ji}). For each pair, we sample one random value from the underlying uniform distribution, which we will call M_{ij}. Then, $C_{ij} = pM_{ij} + (1-p)\bar{C_{ij}}$ and $C_{ji} = pM_{ij} + (1-p)\bar{C_{ji}}$ where $\bar{C_{ij}}$ and $\bar{C_{ji}}$ are new samples from the underlying uniform distribution. When $p = 1$, C is the symmetric matrix given by M, while for $p = 0$, it is fully random.

Non-symmetric Tradeoff: We sample all the off-diagonal elements of C fully randomly from the underlying uniform distribution, and then we constrain all the row sums to be given by $(S-1)C_0 +$

C_d.

Sparse: We randomly select $\lfloor S/3 \rfloor$ off-diagonal entries from each row to be non-zero. Then, we sample these non-zero entries from the underlying uniform distribution.

4.2 Production matrices

Constant: As described in the main text, we set $P_{ij} = 1/(S-1)$ when $i \neq j$ and set $P_{ii} = 0$.

Circulant: We randomly sample a vector of length S from a uniform distribution on $[0, 1]$ and create a circulant matrix by permuting this vector one index as we descend the rows. Then, we set $P_{ii} = 0$ and make the matrix symmetric by taking the Hermitian part. Last, we multiply the matrix by a constant to make all the row sums equal to 1.

Unstructured: We sample all the off-diagonal entries from the uniform distribution on $[0, 1]$ and then enforce that all row sums are 1.

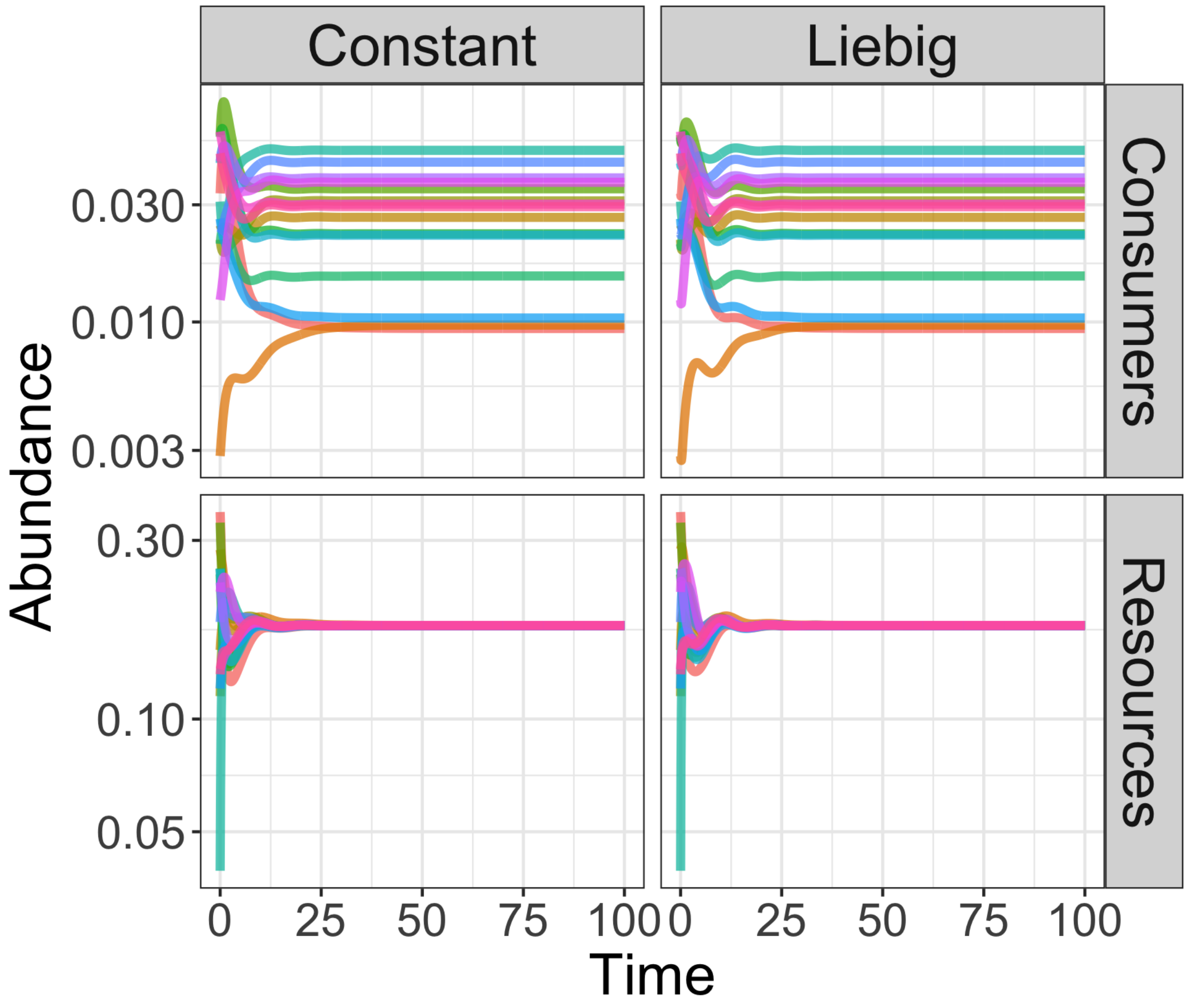

Figure A: **Simplified and Liebig growth rules produce the same equilibria.** We plot the dynamics of consumers and resources (the rows) for two different growth rules (the columns). In the column labeled constant, the consumers never deviate from their assigned resource, while in the column labeled Liebig, they grow according to the Liebig law growth rule described in the main text. After a brief transient where the consumer and resource abundances differ, both of these models reach the same equilibrium abundances. Parameters: $S = 15$, $C_d = 15$, $\eta_i = 1$, $\epsilon = 0.05$ and $\epsilon_{ij} = 1$ for $i \neq j$. ρ is randomly sampled from a uniform distribution on $[0, 0.1]$. The off-diagonal elements of C are sampled from a uniform distribution on $[1, 3]$ and the off-diagonal elements of P and $\tilde{P}$ are sampled uniformly and then the row sums are constrained to be 1.

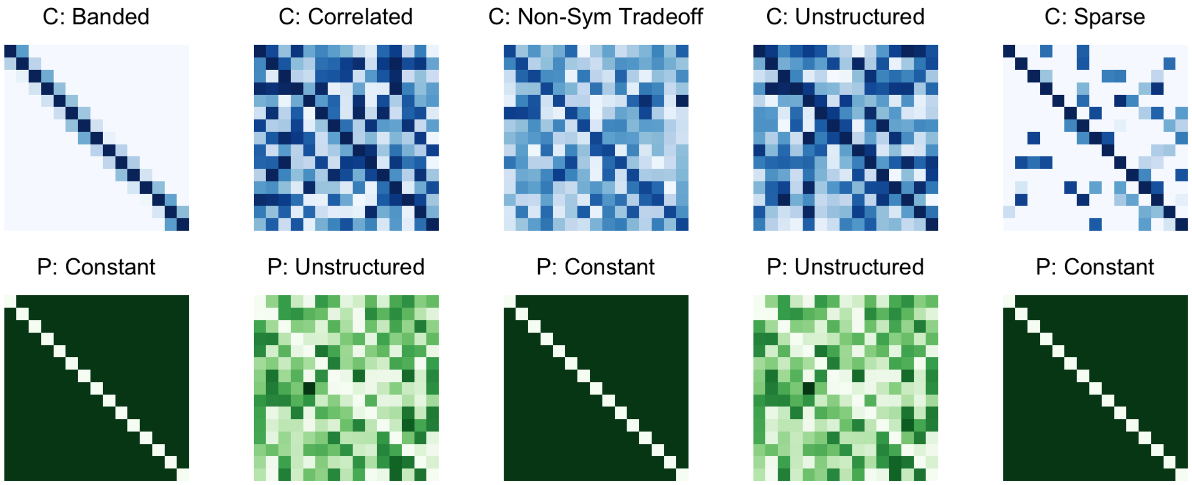

Figure B: **Visualizations of the different matrix parameterizations.** Darker colors indicate larger values. Matrices in the same column are used together to parameterize the system in Fig G, even though we label the heatmaps only by their consumption matrix.

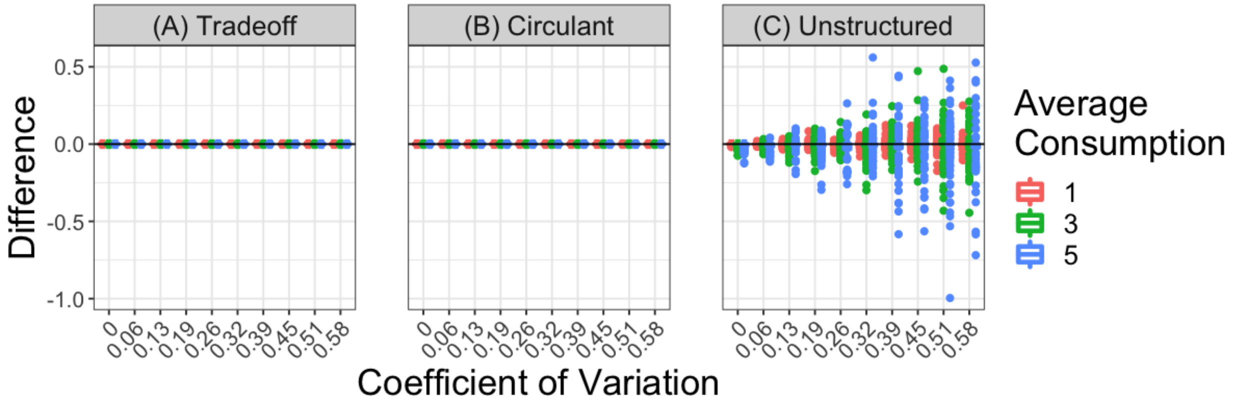

Figure C: **Differences between analytical and numerical C_d values which induce stability.** We plot the difference between the analytical prediction for C_d from the second stability criterion and the smallest value of C_d at which the system becomes stable numerically. We plot the differences as boxplots across a range of different coefficients of variation and three different average consumption values (colors) for the different parameterizations of the consumption and production matrices ((A) is the tradeoff parameterization, (B) is the circulant parameterization and (C) is the unstructured parameterization). The differences are precisely zero when our first criterion is satisfied (panels (A-B)), but in the unstructured case, the difference can be positive or negative. Parameters are the same as in Fig 3 of the main text.

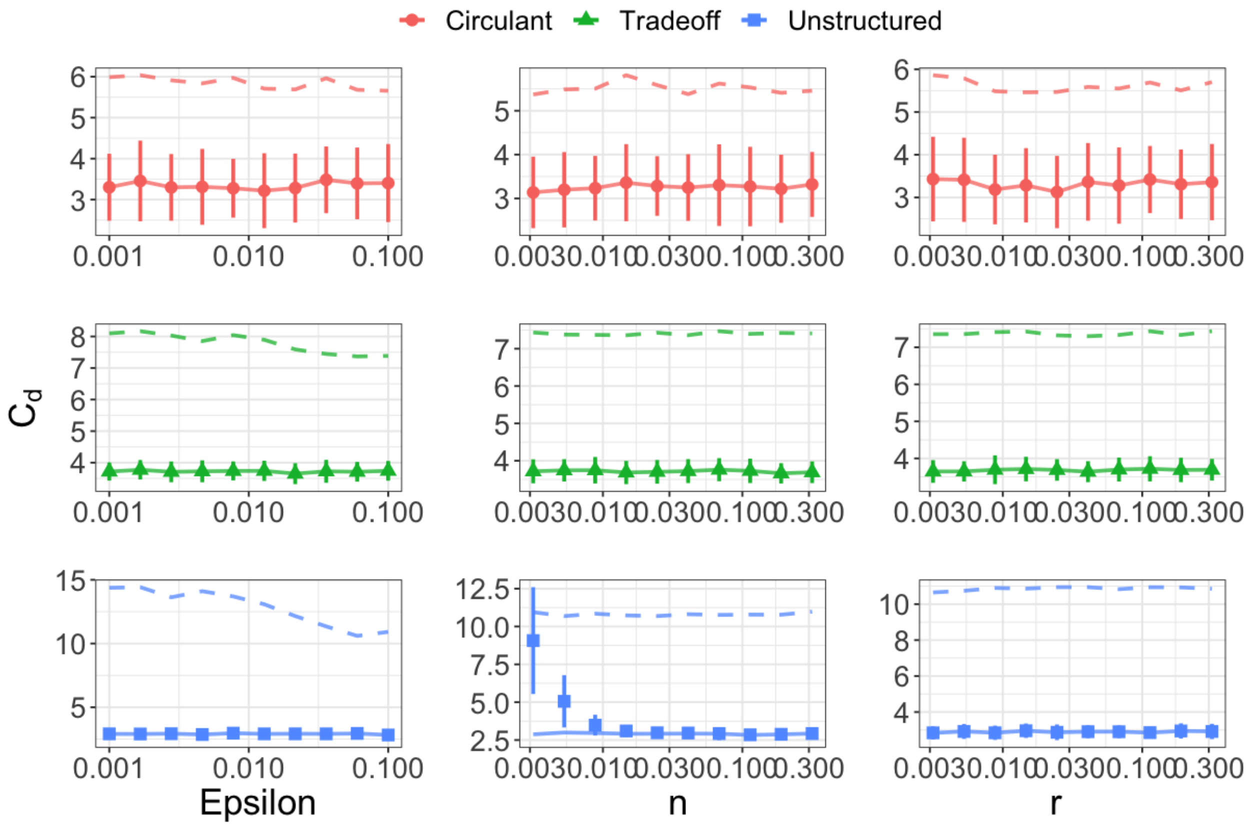

Figure D: **Diagonal consumption C_d required for stability as a function of the parameters ϵ, n and r.** We plot the behavior of the smallest C_d at which B becomes stable (the solid lines) and of the Gershgorin bound on C_d (the dashed lines) as well as the numerically determined C_d at which the system first becomes stable (points and error bars) as a function of the different parameters in the model. We consider the three example consumption and production structures. The rows are the different interaction structures and the columns are the different varying parameters. The middle column (where n is changing) is the same as in the main text. We see that the Gershgorin bound is an overestimate of the numerical C_d values, except for small n in the unstructured case, where it is also violated by the empirical values. In the first column, changing ϵ does not appreciably change the analytical prediction or the empirical C_d values. This is because we are only enforcing stability in these plots which is not affected by ϵ. In the third column, varying r has no affect on the numerically computed thresholds or the analytical bounds for any of the three matrix parameterizations. Parameters: $S = 15$, the off-diagonal coefficients of C are sampled from a uniform distribution on $[0, 2]$ before each of the matrix parameterizations are imposed and $\epsilon_{ij} = 1$ for all $i \neq j$. For the ϵ column, $n = r = 1$. For the n column, $r = 1$ and $\epsilon = 0.05$. For the r column, $n = 1$ and $\epsilon = 0.05$.

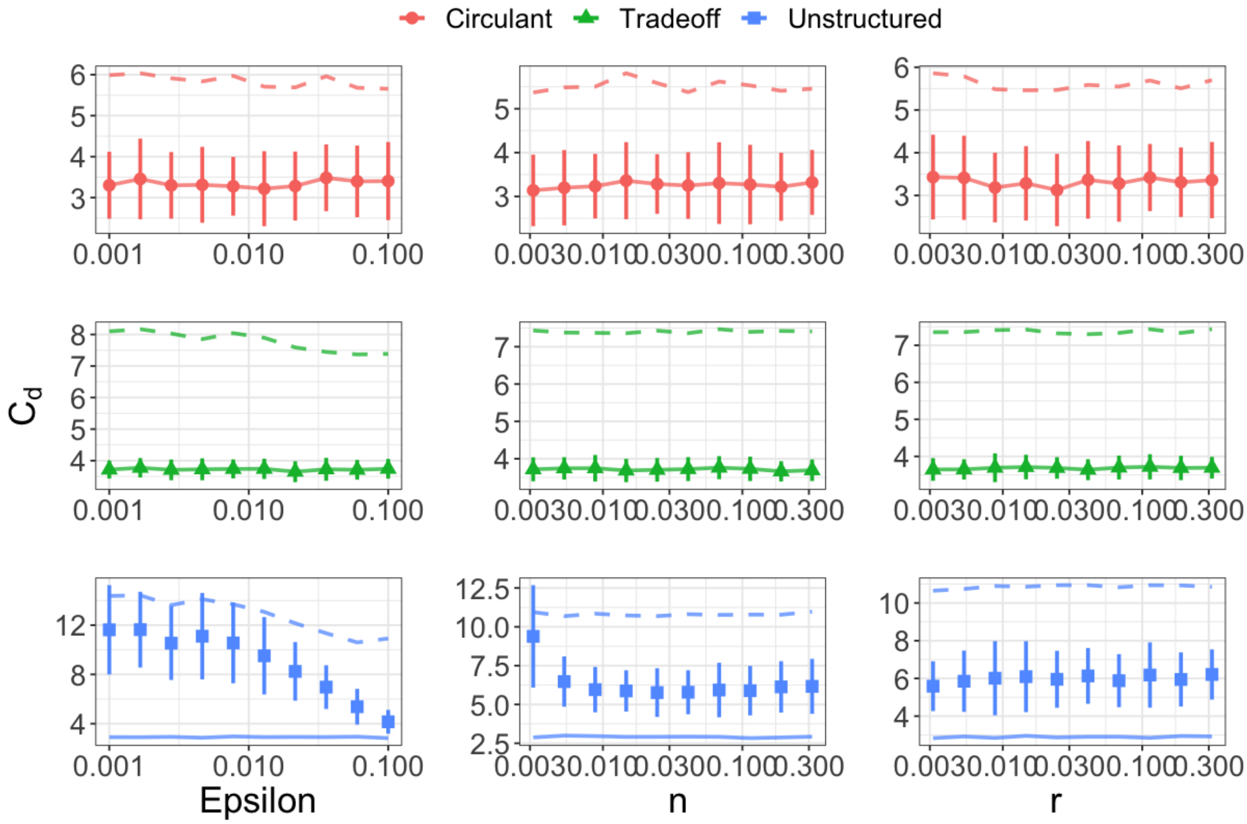

Figure E: **Diagonal consumption C_d required for feasibility and stability as a function of the parameters ϵ, n and r.** We plot the behavior of the smallest C_d at which B stable (the solid lines) and of the Gershgorin bound on C_d (the dashed lines) as well as the numerically determined C_d at which the system first becomes feasible *and* stable (points and error bars) as a function of the different parameters in the model. We consider the three example consumption and production structures. The rows are the different interaction structures and the columns are the different varying parameters. The middle column (where n is changing) is the same as in the main text. The feasibility constraint makes the empirical values of C_d larger than those predicted by the second stability criterion because the system becomes stable before it becomes feasible. The Gershgorin bound, however, is still larger than the empirical values (except for small n values) because it is related to the feasibility criterion. In the first column, changing ϵ affects the Gershgorin bound in all three cases, but it does not appreciably change the empirical values for the circulant or tradeoff cases. In contrast, ϵ does affect the value of C_d in the unstructured case. These behaviors can once again be explained by the relationship between the sufficient stability criterion and the feasibility criteria, since small values of ϵ make feasibility less probable. In the third column, varying r has no affect on the numerically computed thresholds or either of the analytical bounds for any of the three matrix parameterizations. Parameters are the same as in Fig D

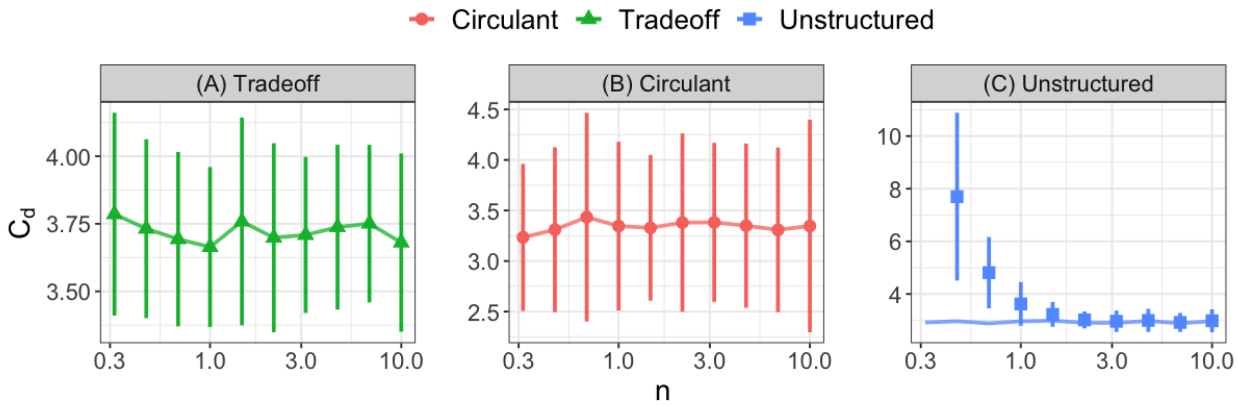

Figure F: C_d **values at which the system first becomes stable for larger** r **values** These plots are the same as Fig 4 of the main text, but now setting r = 125. For these larger r values, the transition to instability occurs at a larger value of the consumer abundances ($n \approx 1$). This effect of the resource abundances r is consistent with our predictions for this threshold consumption values in the no-cross-feeding case.

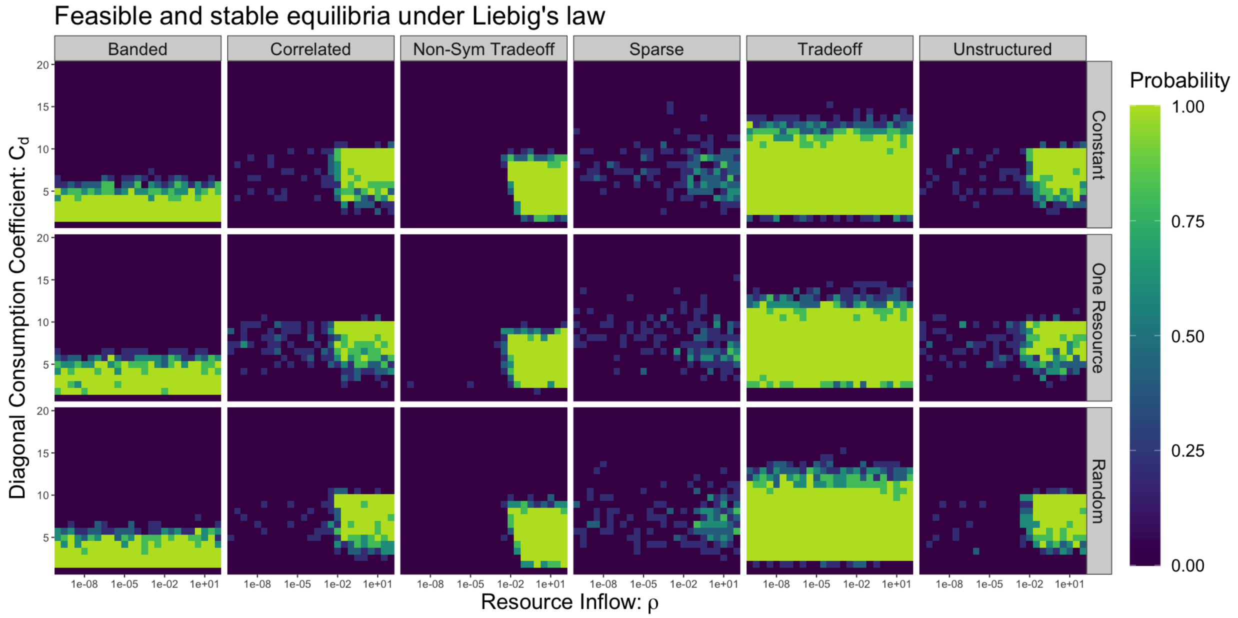

Figure G: **Probability of feasible and stable equilibria under Liebig's law.** We plot the probability of finding a feasible and stable fixed point in 5 replicates across a range of C_d values and resource inflows ρ. We use three different possible resource inflow profiles (the rows). The constant inflow has all resources supplied equally, the one resource inflow supplies only the first resource and the random inflow has all resources supplied at rates sampled from a uniform distribution. In all cases, we ensure that the total resource supply to be given by ρ. We enforce that the fixed point is realized under the Liebig's law dynamics, where each consumer grows on the most limiting nutrient of all the resources. The columns are the different consumption matrix parameterizations, as shown in Fig B. Fig B also shows the corresponding production matrix used to generate the heatmap. As we described in the main text, the matrix parameterizations which generate B matrices with eigenvalues that have non-zero imaginary parts show a transition to instability at low resource inflow. In contrast, parameterizations whose B matrices have purely real eigenvalues do not show this transition. In particular, systems with larger diagonal consumption C_d tend to be stable at lower levels of resource inflow ρ. In each of these cases, the resource inflow profile does not significantly change the probability of feasible and stable fixed points. Parameters: $S = 10$, $\theta = 0.5$, $\epsilon_{ii} = 0.05$, $\epsilon_{ij} = 1$ for $i \neq j$ and consumption coefficients sampled from uniform distributions on $[0.5, 1.5]$ before the constraints are imposed.

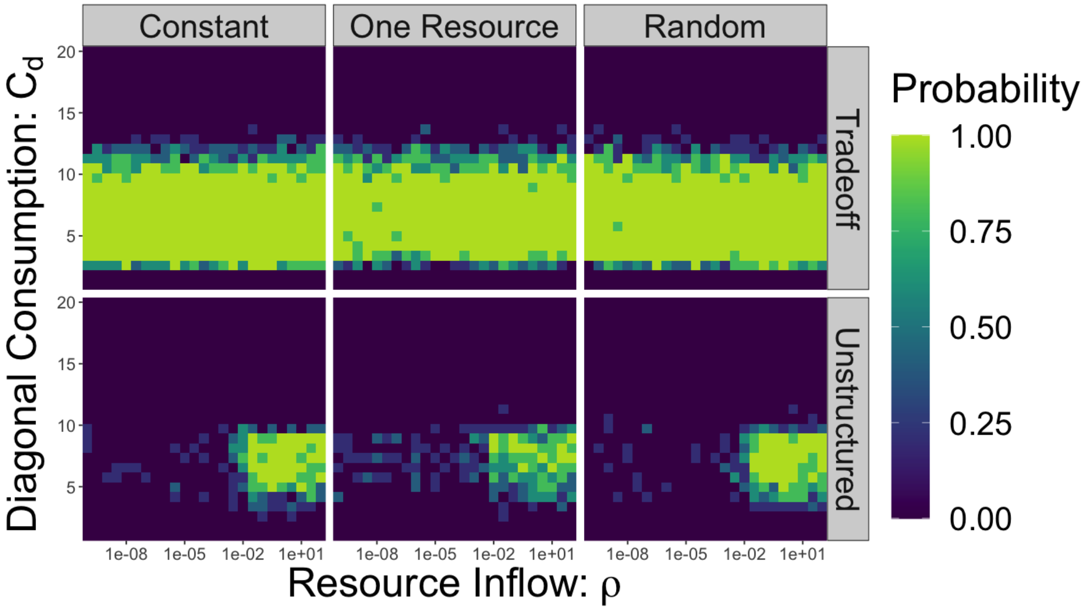

Figure H: **Probability of feasible and stable equilibria under Liebig's law with variable resource abundances.** We plot heatmaps fully analogous to those in Fig G, except we now sample the η_i parameters from a uniform distribution on $[0.9, 1.1]$ so that the resources equilibrium abundances are not all given by the same value. Inflow vector paramterizations are given by the columns while matrix parameterizations are given by the rows. We observe the same qualitative transition into instability as in the main text and Fig G.

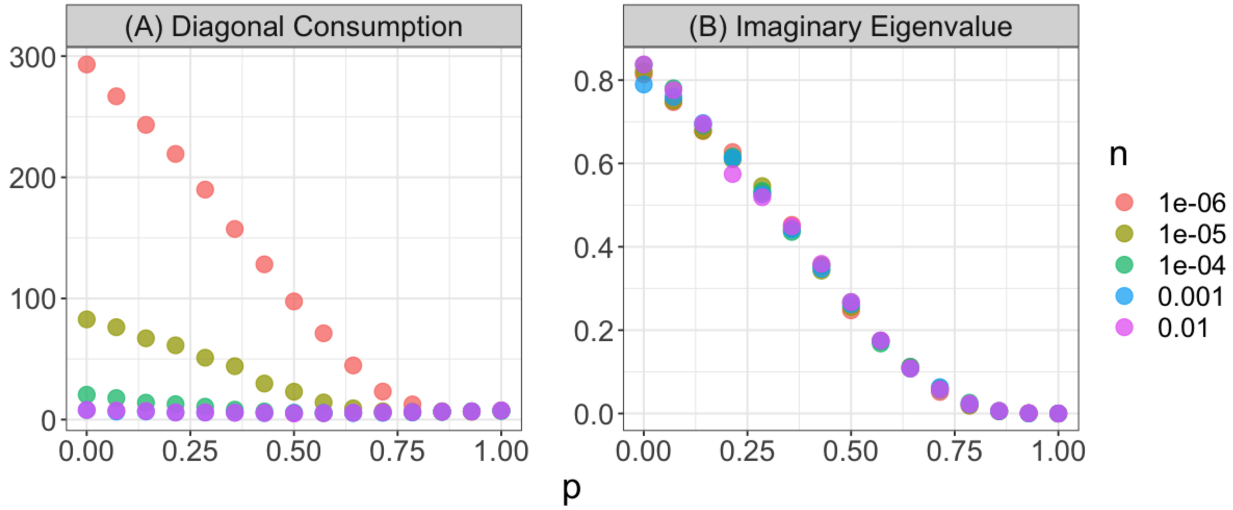

Figure I: **Off-diagonal correlation between the consumption coefficients promotes stability.** (A) We plot the smallest value of C_d at which the system becomes unstable or unfeasible averaged over 5 replicates as a function of the parameter p. In the correlated C matrix case, p determines the correlation between the pair (C_{ij}, C_{ji}) by the formula $C_{ji} = pC_{ij} + (1-p)C'_{ij}$ where C_{ij} and C'_{ji} are uniform random variables on $[0, 1]$. We take P as in the constant case. The different colors are different values of the consumer abundance n. As the consumer abundances decrease, C_d must be large to maintain stability for uncorrelated matrices (ie. small p). However, when p is near 1, and hence the off-diagonal pairs (C_{ij}, C_{ji}) are highly correlated, n no longer affects the numerically determined value of C_d. Because the transition between these two regimes is smooth, even approximate symmetry in the C matrix promotes stability. (B) For the same range of p values and choices of consumer abundance n, we plot the average of the absolute value of the imaginary part of the eigenvalues of the matrix $B = -C + P^T C$. This quantity does not depend on n, but decreases to zero as p goes to 1 when the matrix B has purely real eigenvalues. Parameters: $S = 15$, $r = 1$, $\theta = 0.5$, $\epsilon_{ii} = 0.9$ and $\epsilon_{ij} = 1$ for $i \neq j$.

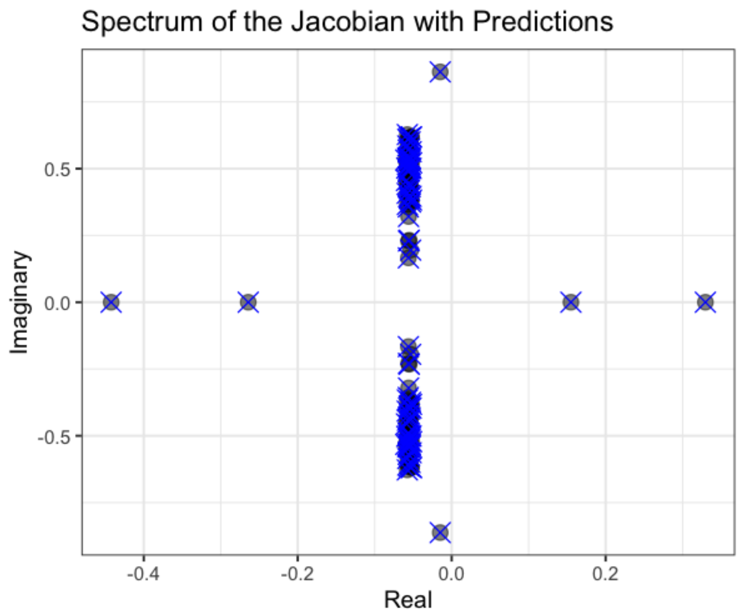

Figure J: **Predicting the spectrum of J from the spectra of A and B when A and B are simulataneously diagonalizable.** Gray circles are the spectrum of J and blue crosses are analytical predictions from the spectra of A and B. Parameters: $S = 100$, $C_d = 10$, $\theta = 0.5$, $\epsilon_{ii} = 0.05$, $\epsilon_{ij} = 1$ for $i \neq j$ $n = 0.001$ and $r = 1$. C and P are given by the circulant parameterization with an underlying uniform distribution on $[0, 2]$ and $[0, 1]$ respectively before the constraints are imposed.

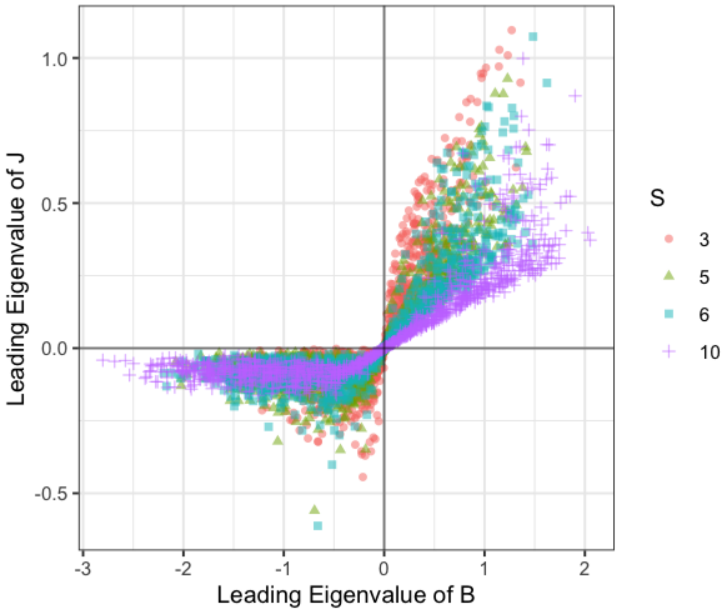

Figure K: J **is stable if and only if** B **is stable in simulations** We plot the eigenvalue of J with maximum real part as a function of the largest eigenvalue of B for 1000 replicates. The different colors and types of points indicate different number of species S. The sign of the leading eigenvalue of B is always the same as the sign of the real part of the leading eigenvalue of J, and the transition from stability to instability (negative to positive values) appears to be sharp.

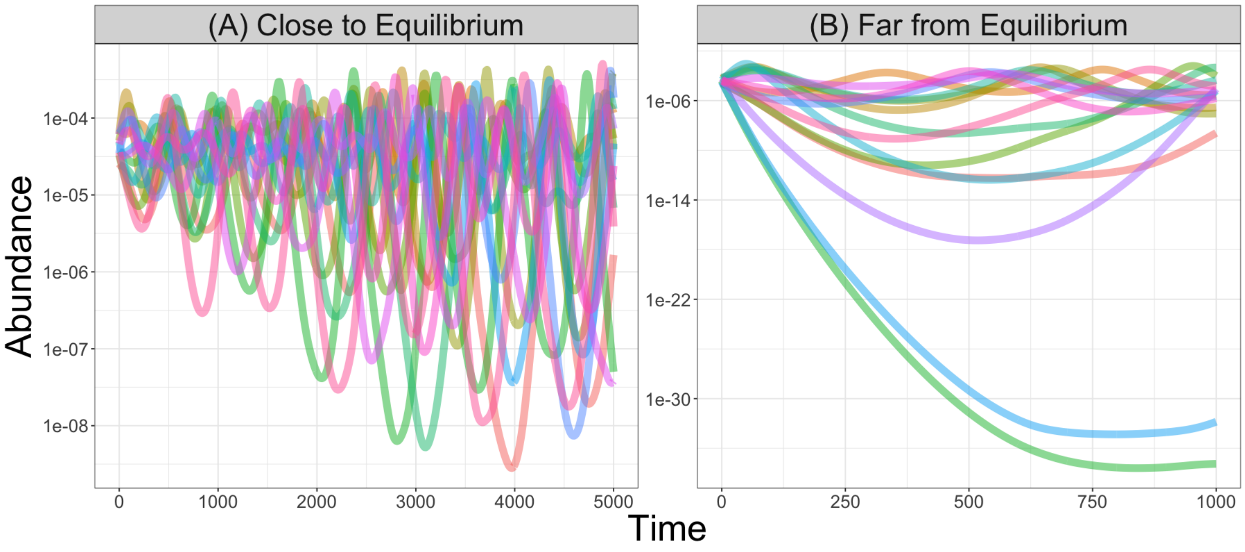

Figure L: **Consumer dynamics close to and far from an unstable equilibrium.** We plot the consumer dynamics over time when the equilibrium is unstable for initial conditions close to (A) and far from (B) equilibrium. To determine the initial conditions, we add noise to the equilibrium values of $\vec{N}$ and $\vec{R}$. We generate $2S$ samples from a normal distribution with mean 0 and a specified standard deviation (0.01 in panel (A) and 0.1 in panel (B)). We then multiply these random samples by the equilibrium abundances and add this quantity to the equilibrium abundances to get the initial conditions. Parameters: $S = 15$, $C_d = 5$, $\theta = 0.5$, $\epsilon_{ii} = 0.05$, $\epsilon_{ij} = 1$ for $i \neq j$ and $\vec{\eta} = \vec{1}$. The C matrices are sampled according to the sparse matrix parameterization with an underlying uniform distribution on $[0.5, 1.5]$. The matrix P is given by the constant parameterization. The resource inflow was $\vec{\rho} = 10^{-4}\vec{1}$. These parameters are the same as those used to generate the unstable dynamics in Fig 1 of the main text. The stable dynamics in Fig 1 are also the same parameters except with the total resource inflow $\rho = 1$.

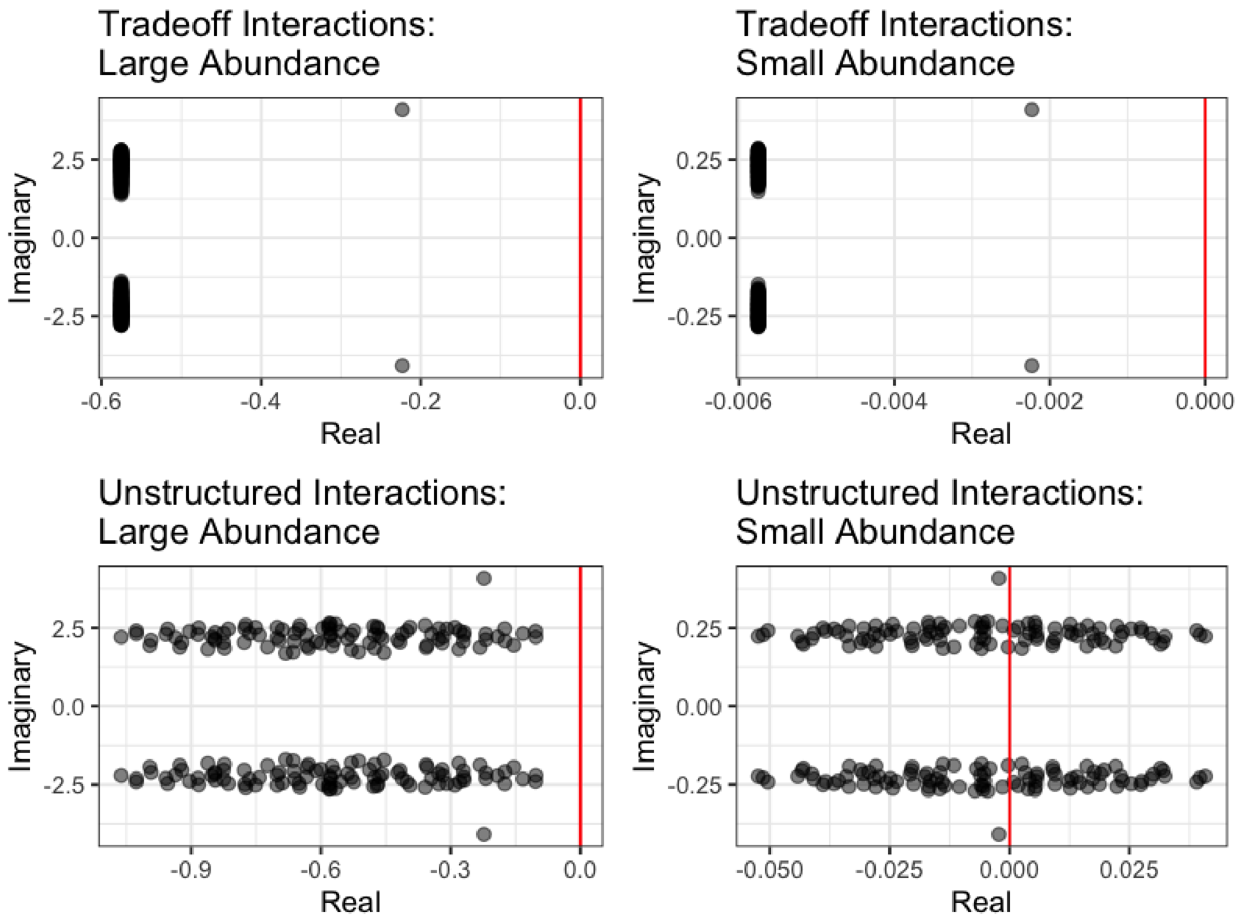

Figure M: **The spectrum of J for small and large consumer abundances.** We plot the spectrum of J when the consumer abundances are small $n = 0.0001$ and large $n = 0.01$ under the tradeoff and random parameterizations. Red vertical lines indicate the imaginary axis. In all four cases, there are two bulks of eigenvalues, which are each a transformed version of the spectrum of B. When the consumer abundances are small, these two eigenvalue bulks appear as two clouds of eigenvalues with non-zero imaginary parts for both parameterizations. For the tradeoff parameterization, the clouds have no width, because the underlying B matrix has purely real eigenvalues. For the random parameterization, the clouds have non-zero width because B has eigenvalues with non-zero imaginary part. As the consumer abundances become small, the non-zero width of these clouds means that an eigenvalue complex conjugate pair eventually crosses the imaginary axis at a value $n > 0$. Parameters: $S = 100$, $C_d = 15$, $\theta = 0.5$, $\epsilon = 0.05$, $\epsilon_{ij} = 1$ for $i \neq j$ and $r = 1$. In each of the parameterizations, the consumption coefficients are sampled from a uniform distribution on $[0, 2]$ before the constraints are imposed. In the tradeoff parameterization, the production matrix is constant, while in the random parameterization, the production coefficients are sampled from a uniform distribution on $[0, 1]$ before the constraints are imposed.

References

[1] Horn, Roger A. and Johnson, Charles R. *Matrix Analysis*. Cambridge University Press, New York, NY, second edition, corrected reprint edition, 2017. ISBN 978-0-521-54823-6 978-0-521-83940-2.

[2] Kermorvant, Claire and D'amico, Frank and Bru, Noëlle and Caill-Milly, Nathalie and Robertson, Blair Spatially balanced sampling designs for environmental surveys. Environmental monitoring and assessment. 2019;191(8):1-7.
