# Supplementary material for: Stability criteria for the consumption and exchange of essential resources
Source: PLoS Comput Biol. 2022 Sep 8;18(9):e1010521. doi: 10.1371/journal.pcbi.1010521 (PMC9488833; doi:10.1371/journal.pcbi.1010521)
Supplement: Attachment — Submitted filename: essential-resources-reviewer_responses.pdf [file pcbi.1010521.s002.pdf]

The reviewers raised substantial criticisms on the organization of the manuscript and its clarity, which makes it hard to judge the biological significance of the model assumptions. If you decide to submit a revised version, it is important that you address all of the reviewers' concerns. This would make the manuscript much more accessible to the broad audience of Plos CB

We thank all three reviewers for their suggestions and criticisms. Addressing them has greatly improved our manuscript. We believe the reviewer’s comments can be grouped into two main categories.

First, the reviewers questioned whether our modeling assumptions were biologically realistic. To address this, we have modified the model in our revised manuscript to incorporate more biological realism. In our new model, non-limiting resources now contribute to biomass. We have added a new conceptual figure (Fig. 1(A) in the manuscript) to display the flow of resources through a single consumer, and we copy this figure below. As before, consumers deplete two types of resources – one that limits their growth and all the other resources that they deplete. The limiting resource goes purely towards satisfying their internal requirements. Due to inefficiency in the processes that build biomass, some of this limiting resource can be cross-fed as byproducts, but the rest goes towards microbial growth. For the non-limiting resources, consumers will have depleted them in excess of their requirements for growth. This excess resource is leaked back out into the environment, possibly in the form of other resources. The remaining internal resource concentrations are now used to satisfy the consumer’s requirements, and so they are treated in the same way as the limiting resource contributes towards organism requirements. Overall, these processes mean that consumers use resources in fixed proportions to build biomass. We describe this new model form in detail in lines 74-145 with the aid of two conceptual figures. In the revised manuscript, we show that our previous theory can be generalized to accommodate this more realistic model, suggesting that our stability criteria apply to a wider range of models than we have explicitly considered here, as long as consumers have a specific limiting resource.

Second, the reviewers found the manuscript to be overly complicated. We have made substantive edits which we believe have clarified the presentation of our results. We moved large portions of the more involved mathematical discussion to the Appendix. We have also more clearly designated our four main results in the Results section and in a summarizing paragraph in the Discussion. Throughout the main text, we attempted to simplify the presentation of our mathematical and numerical analyses and focus on four key takeaways.

Please see our point-by-point responses to the reviewer comments below, where we also address issues that do not cleanly fall into the two categories above.

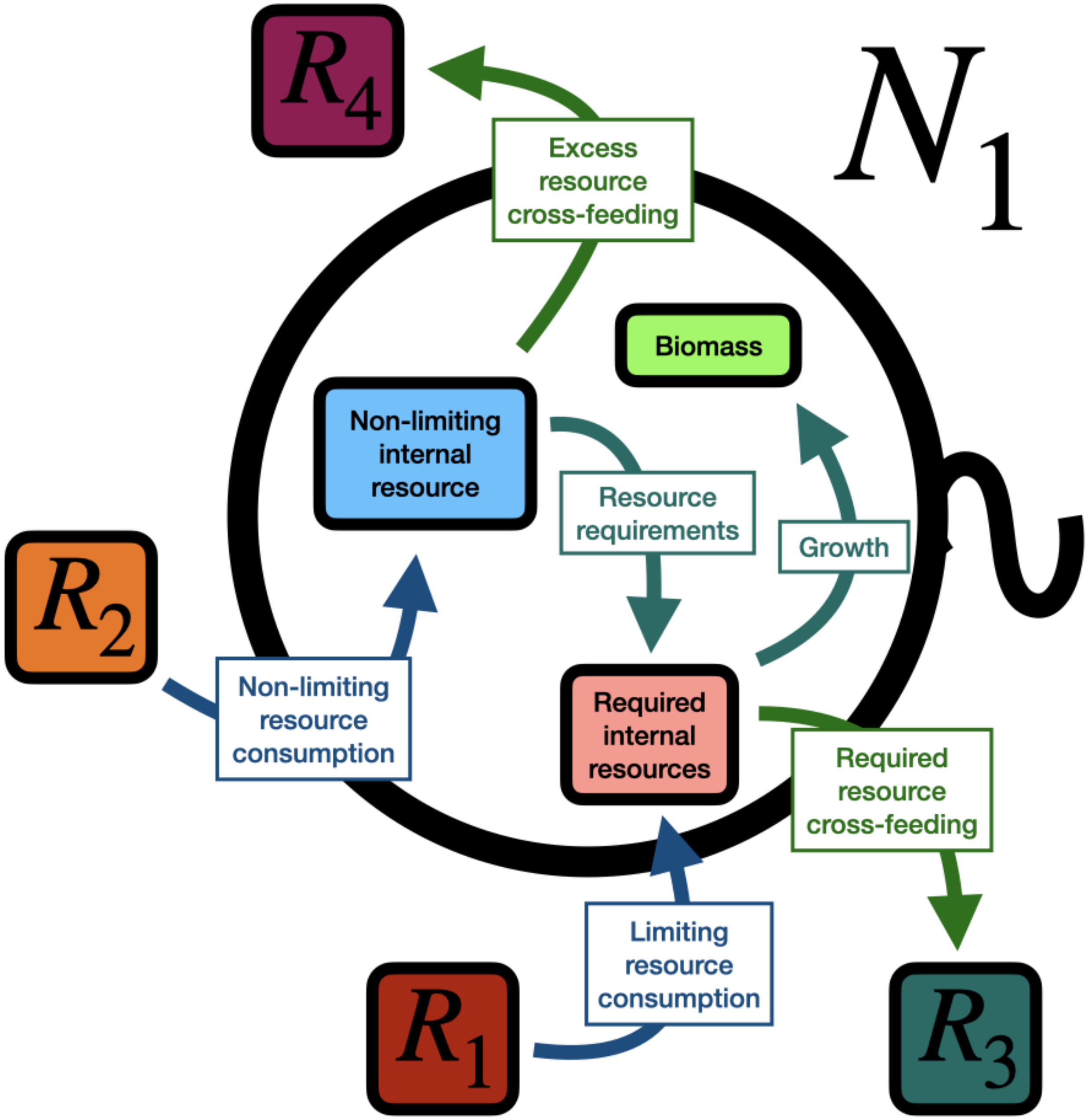

Reviewer's Responses to Questions

Comments to the Authors:

Please note here if the review is uploaded as an attachment.

Reviewer #1: The paper extends consumer resource models with cross-feeding interactions to incorporate Liebig's law. The authors propose two stability criteria and find that the model exhibits chaotic behavior at low consumer abundances. I find the chaotic behavior very interesting, but I am very concerned about its biological significance.

We are glad that the reviewer finds the chaotic behavior interesting. In our responses below, we aim to more clearly outline the biological significance of our modeling. As we described above, we have now modified our modeling framework to be more biologically realistic.

1. The current form focuses on stability analysis or chaotic behavior, fitting more to a theoretical biology journal.

We agree with the reviewer that our focus in this paper is on stable coexistence. Specifically, we aim to determine when multiple species may stably coexist on limiting resources. In addition, these species interact through both competitive and cooperative interactions. We believe our results will be of interest to the diverse readership of PLOS Computational Biology because we use a combination of mathematical and computational approaches to identify patterns in the interactions of microbial communities that can be tested experimentally.

2. As noted by the authors, the stability and feasibility analyses require restrictive assumptions, which are hard to fulfill in real ecosystems. In the main results, eq. (3) ignores the difficulty of finding the feasible solution satisfying all consumer abundances positive. While eq. (4) is conditioned that all consumer abundances are identical, which requires resource supplied rates and the row sum of the consumer matrix are equal, respectively.

We agree with the reviewer that Eq. (3) alone does not guarantee that the consumer abundances are feasible. In lines 176-189 following Eq. (3), we describe the various constraints that need to be satisfied for the abundances to be positive. In Fig. D and E of the Appendix, we plot two versions of Fig. 4 — one where only stability is enforced and one where feasibility and stability is enforced. We describe in detail how enforcing feasibility changes these plots in the “Feasibility analysis” section and figure captions of

the Appendix, but when consumer abundances are small, stability determines the C_d values in our simulations, as predicted by our theory.

The reviewer correctly points out that in Eq. (4) we assume that all species have equal abundances, which in turn requires the resource inflow rates and the row sums of the consumption coefficients to be carefully chosen. We derive our analytical theory in the simple setting in which all consumer abundances are equal, but, in our third main result, we show using simulations that the qualitative results of our theory generalize to cases where consumer abundances are not identical (Fig. 5). The "Feasibility Analysis" section of the Appendix also includes a more detailed analysis of the feasibility of the equilibria in our model. We show, for example, that our two stability criteria and the feasibility of the equilibrium are related.

I can not recommend the paper to publish on PLOS Computational Biology for all the above reasons. I also have some comments that could be useful if the authors decide to work more on the chaotic dynamics.

1. The authors do not distinguish between unstable and chaotic dynamics. The instability is the necessary condition for chaotic behaviors. Appendix 1.5 shows the theoretical proof for the case without cross-feeding. However, in the limit n->0, the resources undergo unbounded growth, which is unstable but not chaotic.

Our focus in this paper has been to establish under what circumstances feasible solutions exist and are stable. The subsequent dynamics following the departure from an unstable equilibrium could indeed be chaotic, but as the reviewer points out our instabilities may just as easily lead to non-chaotic dynamics. We agree that an important subsequent question is to determine the circumstances under which the dynamics are chaotic. But we also think it is important to establish when equilibria are stable, which has not yet been fully explored in models with cross-feeding. When we discuss the limit of low consumer abundances, we do not set the consumer abundances to zero exactly – we just make them small. As a result, there is always a non-zero density of consumers in the system which can deplete the available resources, meaning that resource abundances do not diverge. More generally, we simulate the dynamics in Fig. 6 (as well as SI Fig. K) and observe that there are a variety of complicated behaviors that can occur, depending on the stability of the equilibrium and the initial conditions.

2. It would be helpful if the authors could clearly explain the origin of chaotic dynamics. I guess it results from random switches between different resource preferences, and thus eq. (2) should not exhibit chaotic behaviors. It is easy to check it numerically.

Interestingly, the unstable dynamics can occur in the ODEs in Eq. (2), meaning that the resources switching mechanism is not required for these dynamics. At the same time, we do not have definitive proof that these dynamics are indeed chaotic – it is possible that they are complicated limit cycles with a long period. As a result, we focus on the transition from stability to instability because we have an analytical understanding of this behavior that aligns with our numerical evidence. Additionally, because the unstable dynamics typically include fluctuations to very small densities, we would not expect these dynamics to persist in natural communities regardless of whether or not they are truly chaotic.

3. In principle, the population abundance can be rescaled to different values by tuning model parameters. If the chaotic behavior is general, it should not exhibit only "at low consumer abundance." At least, it is possible to show the chaotic dynamics in order ~ 1. Otherwise, we cannot rule out the possibility of numerical instability in the ODE solver when the values are small.

We thank the reviewer for this suggestion. In our theoretical analysis of both the no-cross-feeding case and the simultaneously diagonalizable C and P matrices, we can explicitly derive the value of the consumer abundances n at which the system becomes stable. For example, in these simplified models, as the resource abundances r increase, the smallest value of n for which the unstructured network becomes unstable decreases. In the plot below, we recreate our Fig. 3, but now setting r = 125, and observe that the transition to instability now occurs for n values around 1. Note that, in this figure, we cannot derive the exact threshold for instability, but the qualitative lessons we derived from the simpler models still apply. In other words, we can choose parameters appropriately so that exceptionally small consumer abundances are not required for instability. This suggests that the instability we have identified is not due to a numerical instability in the ODE solver. Moreover, in SI Fig. L, we plot the spectrum of the Jacobian matrix and observe that some eigenvalues cross the imaginary axis when n is small, producing an unstable fixed point. Overall, the fact that our simulation results corroborate our analytical predictions is very unlikely to result from numerical stability.

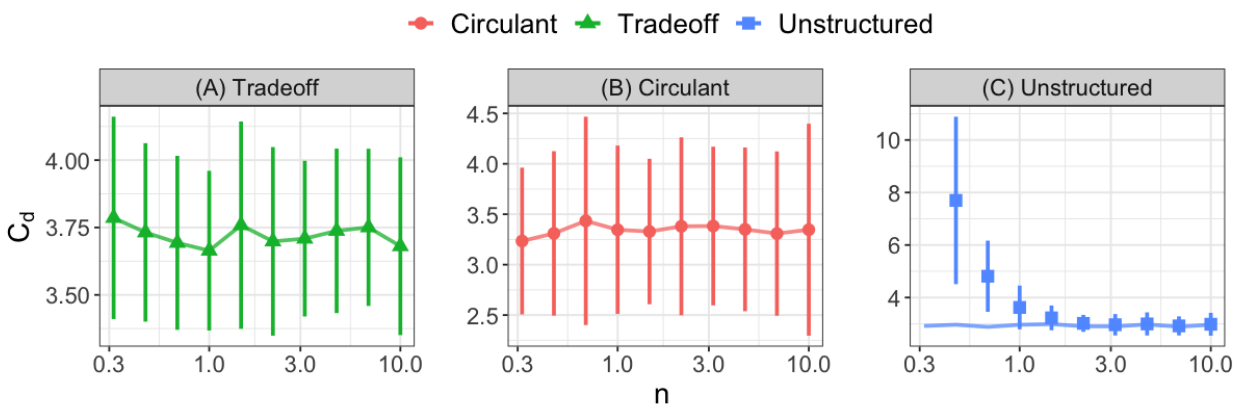

Reviewer #2: This manuscript develops stability criteria for complex microbial communities that interact through resource competition and cross-feeding. Unlike previous related work, this study focuses on essential resources. The manuscript combines elegant analytical results from matrix theory with numerical simulations to flesh out the conditions for stability. Cross-feeding is common in microbes but relatively understudied, so this is a timely and important topic. I believe the manuscript will be ultimately publishable, but have some questions about the model formulation and suggestions to improve the clarity.

We appreciate the reviewer's positive opinion of our work. In our revised manuscript, we have attempted to modify the model formulation so that it is more biologically realistic and simplify the presentation of our results.

1) I was a bit unsure about the formulation of essential resources using Liebig's law of the minimum. In the usual interpretation, all resources contribute to production of new biomass in a fixed ratio. By definition, non-limiting essential resources don't LIMIT growth, but are still REQUIRED for growth. However the authors interpret this as "each consumer can only produce new biomass using one resource at a time ... we define the function g_k(R) to choose which resource the k-th consumer grows on" (lines 82-84). A consequence of this assumption is that whatever resources a consumer does not "grow on" have delta_jk=0, so in eq. 1 we see that they are completely excreted by the consumer. It seems that after excreting all of the non-limiting resources, none will remain for growth requirements so they actually should become limiting. Previous work on resource excretion takes into account this need for balance (see e.g. Sterner 1997 Freshwater Biol. 38: 473-481). Thus the formulation does not line up with how essential resources are commonly considered, unless I'm not following the argument.

We thank the reviewer for this important point. In the revised manuscript, we have modified our modeling framework to include a more realistic version of Liebig's law.

There are two possible paths for depleted resources through a consumer. For the limiting resource, the entirety of the depleted abundance goes towards the consumer's growth requirements. Some portion of this resource is still cross-fed back into the community as a result of the internal processes that build biomass. We interpret this as an inefficiency due to the permeability of cell membranes, but there may be evolutionary reasons for this leakage as well [1]. The remaining portion goes towards microbial growth. For the non-limiting resources, consumers will have depleted the resources in excess of their minimum requirements. The portion of the depleted, but non-limiting, resources that are required for growth undergo the exact same process as the limiting resource we described above. The excess resource – that is, the consumed resource that is above the microbe's minimum requirements – is all leaked back into the environment, possibly in the form of new resources. Overall, these processes mean that consumers use resources in fixed proportions to build biomass, exactly as described by the reviewer. We describe this new modeling framework in detail in lines 74-145.

2) The authors consider both stability and feasibility of coexistence equilibria, but not uniqueness. It is possible in cross-feeding interactions to have multiple coexistence equilibria, some stable, some unstable (Sun et al. 2019 JTB 465: 63-77). Therefore if one equilibrium is unstable, it does not necessarily mean that the species can't coexist. Is this a possibility in the authors' model?

We thank the reviewer for raising this question. There is a single unique positive solution to Eq. (3), so for a given assignment of limiting resources to consumers, there is only one coexistence equilibrium. It is possible, in principle, that our Liebig's law growth rule could generate multiple different assignments of consumers to resources, which then might produce multiple equilibria. However, we never observe this behavior in simulations. Instead, there is some transient switching between consumer-resource pairs but the Liebig's law model eventually converges to the same equilibrium as the model where consumers do not change their limiting resources (SI Fig. A).

For different growth rules or in different parameter regimes, it is certainly possible for there to be multiple equilibria. In this case, our theoretical analysis would still apply to these multiple equilibria (in a local neighborhood of each). There could also be continuous switching between limiting consumer-resource pairs, in which case our analysis would no longer apply, because any coexistence in this situation would be out-of-equilibrium. We describe the possibility of non-equilibrium coexistence in lines 506-511 of the Discussion, and we have added a citation to the work that the reviewer pointed out and a brief discussion of the possibility of multiple equilibria.

The manuscript is complex, so the authors should do more to help readers. Here are some places I was confused. With sufficient effort I could figure these out, but I think the authors should make it easier on the reader.

We thank the reviewer for this suggestion. We have attempted to simplify the presentation of our results throughout the manuscript. We discuss many specific changes in our responses below.

Some other major changes include: In lines 74-145, we describe our new model with the aid of an additional conceptual figure. We believe this new figure will help clarify our model assumptions and aid readers in visualizing the model. Throughout the Results section, we have also added summarizing sentences to clearly highlight each of our main results. In the second paragraph of the discussion, we summarize all four of these main results (lines 408-426). We also removed sections of the Discussion that were overly technical or mathematically involved. We hope that these edits will make the manuscript easier to read.

3) In the statement of the two stability criteria in lines 177-178, it is not clear whether these two criteria BOTH need to be satisfied to guarantee stability or EITHER suffices. It's also not clear whether these are necessary or merely sufficient. Since this is a central result that structures much of the rest of the paper, this should be made obvious.

We thank the reviewer for pointing this out and we have added a clarifying sentence before the stability criteria (lines 199-200). Both of these criteria need to be satisfied for stability to be guaranteed. In other words, together they are sufficient for stability. They are not necessary, as the unstructured case (which does not satisfy our first stability criterion) can still be stable (Fig. 3-5). Last, we have multiple lines of analytical and numerical evidence that if our first stability criterion is satisfied, then the second criterion is sufficient *and* necessary for stability of the whole system, as we lay out in lines 281-290.

4) The description of choices of interaction matrices was confusing. Line 236 says that THREE interaction matrix structures will be studied, line 238 says we consider TWO different methods of parameterizing the matrices, see Fig. 2, which has FOUR matrices. This could use more careful exposition.

We thank the reviewer for pointing this possible source of confusion. We have significantly edited this section and merged it with the previous subsection titled “Sufficient stability criteria for constant abundances.” We now lay out the two

consumption and production structures that satisfy our symmetry constraints and the “unstructured” case which does not in lines 242-264.

5) In writing my review, I thought I'd summarize the main findings, but I realized I wasn't sure what they were. The abstract says "We identify special consumption and production networks which protect the community from instability when resources are scarce". The discussion says "We identified a set of symmetric consumption and production networks that guarantee stability as long as intra-specific regulation is large compared to inter-specific interactions" (lines 475-477). What are those networks that promote stability? What are the key parameters that affect stability and how? Those should be the main conclusions, but they're not easy to find. The take-away messages should be made obvious in the abstract and highlighted in the beginning of the discussion.

In our re-writing of the Results and Discussion sections, we have attempted to make our main results more apparent. Each time we describe one of the main takeaways in the Results section, we now summarize it in an additional sentence (for example, lines 290-293). We have also introduced a summary of these four main results (lines 408-426) in the second paragraph of the Discussion:

“First, our two stability criteria were not only sufficient for, but in fact appear exactly predictive of, stability in simulated communities. Second, reciprocal interaction structures protected the community from instability at low consumer abundances, but communities with non-symmetric interactions generically became unstable when resources were scarce. Third, our analytical results applied qualitatively to situations where microbes compete for a single external resource and therefore have highly variable consumer abundances at equilibrium. Fourth, interaction networks that were not exactly symmetric still promoted stability, as long as the spectra of these networks had small imaginary parts.”

Some other stylistic suggestions:

6) Make a table of key variables and be more liberal in using verbal descriptions of variables in the text.

We thank the reviewer for this suggestion. We have inserted a table of the mathematical variables following the model equations (Table 1) and we have added descriptions to many of the variables we mention in the main text in addition to mathematical symbols.

7) Don't refer just to "S1 Appendix", give specific parts so readers can find the relevant section.

We have now added these specific references.

Reviewer #3: The authors model an arbitrarily large number of microbial species feeding on an equal number of resources. The chemostat model includes cross-feeding, or the use by one organism of metabolites released by other organisms. The authors find conditions for feasibility and stability of the system that depend on structural aspects of the system.

Comments

Figure 1. It appears that the red arrows mean that the consumer that die are removed from the system, as washout, and that is reflected by eta*N in equation (1). But there is no similar washout of the resources. Is that because uptake rates are assumed much higher than washout rates of resources?.

Yes, that is correct. We have added a sentence to the description of our model explicitly acknowledging this assumption (lines 106-108).

As the authors note, their model has some things in common with the well known models based on zero net growth isoclines (ZNGIs) and follow the general rules of those models regarding stability; each consumer being limited by one resource, each species regulating the resource that limits it more than any other resource, and the supply point being above the ZNGIs. I am guessing that the resources then are inorganic and/or organic compounds that enter the chemostat are taken up and either used to build biomass (only one of them) and the others released back into the environment. The authors make some additional assumptions.

The reviewer is correct in their interpretation of our model.

"… each consumer can only produce new biomass using one resource at a time (which we define as the limiting resource) … This is a restrictive assumption, since some bacteria may incorporate biomass from non-limiting resources in nature." (Lines 82-86) The last sentence sounds like an understatement to me, as I would have assumed that in general bacteria would be taking up resources other than its limiting resource. Is there some empirical evidence that the assumption being made here is even approximately reasonable?

The reviewer is correct that the assumption that the consumption of non-limiting resources do not contribute to biomass was severe. We thank the reviewers for identifying this unrealistic feature of our model. We have now modified this assumption so that consumption of all resources contribute to biomass, and only the excess, non-limiting resources are directly cross-fed (byproducts from the processes that produce biomass may also contribute to cross-feeding). We describe our modifications in detail in the summary of all of our responses (above), our responses to reviewer #2, and in lines 74-145 of the manuscript.

Lines 131-132. “each resource is limiting for precisely one consumer because of the competitive exclusion principle.” That perhaps shouldn’t be stated so absolutely, a co-limitation seems to be common, as least in primary producer communities; e.g. Harpole, W.S., Ngai, J.T., Cleland, E.E., Seabloom, E.W., Borer, E.T., Bracken, M.E., Elser, J.J., Gruner, D.S., Hillebrand, H., Shurin, J.B. and Smith, J.E., 2011. Nutrient co‐limitation of primary producer communities. Ecology letters, 14(9), pp.852-862. Clearly, of course, the assumption of one limiting resource for each consumer makes equation (2) and analysis much simpler.

We thank the reviewer for this comment. We agree that nutrient co-limitation is common in nature. We meant to argue that *two different consumers cannot coexist on one resource in our modeling framework.* We now more carefully lay out the logic of this statement in the first paragraph of the subsection labeled “Equilibria of the consumer-resource model” (lines 147-157). In this paragraph, we explicitly note the possibility of nutrient co-limitation and cite the reference above.

Lines 187-190. “Consumer i grows only on resource i, so consumer j’s effect on resource i (and hence consumers i’s growth) is exactly matched by consumer i’s effect on resource j. Although we don’t expect natural systems to be precisely symmetric….” (This sentence is repeated almost word for word in lines 404-406.)
Again, the second sentence sounds like an understatement. I don’t see why that sort of symmetry, or reciprocity of interactions, should even approximately occur. If it does, it is very interesting, but it would be good to see at least some evidence that this might be roughly a pattern in nature. I realize this is necessary for matrix B to be symmetric, which then of course has the very nice property of negative eigenvalues. So it is an interesting idea that bacterial systems could evolve toward this sort of situation, where they are stable.

We thank the reviewer for pointing out the duplicate phrasing and we have removed the sentence in the results. We agree that reciprocal interactions may not be the norm in most natural microbial communities. At the same time, we also agree with the reviewer

that microbial ecosystems could potentially evolve towards this limit, though this is beyond the scope of the current work. Importantly, we showed in simulations that interaction networks which only approximately follow our symmetry criteria are still stabilized relative to interaction networks that are less symmetric (see Fig. 5 and SI Fig. H). In our revised manuscript, we now describe this as our fourth and final main result (lines 371-377 and 425-426). Specifically, interaction networks with eigenvalues that had small imaginary parts were more likely to be stable than those with large imaginary parts (SI Fig. H). In other words, we related our symmetric stability criteria to a general property of all interaction networks (namely, the spectrum of the network). We then showed that the spectrum of this network influences the stability of the system as a whole. Because the imaginary parts of the eigenvalues of the B matrix can be computed for any interaction network, we can evaluate the extent to which an arbitrary network is stabilized by being near to our symmetry criterion. We have not yet fully characterized the effect of these spectral properties on stability. Nevertheless, our results suggest that a quantitative generalization of this strict symmetry condition may hold. In general, we believe that an exciting direction for future work is to understand how the empirical structure of microbial interactions affects coexistence, and these results represent first steps in that direction.

To this end, we note two possible forces that could structure microbial interactions besides evolution. First, it is possible that the process of community assembly itself introduces constraints on the kinds of networks that a coexisting community can have. For example, recent simulations of a related consumer-resource model [2] found that microbial species tended to interact in a characteristic pattern when resources were scarce. We describe this connection in lines 453-458 of the Discussion. Second, the cross-feeding of resources in nature has to obey biochemical constraints that we have not modeled explicitly. Similarly, there are well-characterized structures in metabolite leakage networks that have been shown to describe the composition of experimental microbial communities [3]. We briefly discuss how these structures might connect to the symmetric structures we identified in lines 435-442. All in all, we do not yet know whether or not the symmetry conditions we found analytically are present in real microbial ecosystems, but our findings suggest future research in this area, and suggest concrete hypotheses for experimental studies.

Line 204. “satisfying Eq. 4 ensures that each consumer regulates its own resource more strongly than other consumers affect it”
This calls to mind that another approach to large systems, in this case host-microbe systems, is that of James Bever and colleagues; e.g., Bever, Westover, and Antonovics 1997; Eppinga et al. 2018. The modeling of Bever and colleagues is different in that it deals with hosts (plants) and their microbial symbionts, which may be pathogens or

mutualists, rather than consumers and resources in the present case. However, some aspects are similar, such that it deals with large systems, that overall self-limitation of individual species, or negative feedback at the system level leads to stability, and that there are two types of entities, unlike Lotka-Volterra competition models. A difference is that the Bever et al has models have actually been used successfully to interpret empirical data.

We thank the reviewer for pointing out this connection to the work of Bever and others. We now include a discussion of the relationship between our work and this previous theory in lines 234-237: “This mirrors classic results for consumer-resource models with two resources [18], negative feedback in the theory of plant communities [32, 33] and the central role of self-regulation in multi-species stability theory [2, 3].”

Line 221 “the second criterion ensures that the strength of the inter-species interactions is not too large relative to the inter-species interactions in the community”. This is somewhat trivial as it is well known. Why investigate this?

In the consumer-resource model we consider, it is difficult to identify the strength of inter-species interactions in the first place, because our model does not contain these types of interactions as inherent parameters. Instead, the effective interaction matrix B emerges non-trivially from our mathematical analysis. In other words, one of our main results is that the B matrix can both be interpreted as the effective interactions between consumers and is predictive of stability. Although our results do not always align with the theory of pairwise models, our second stability condition is one important connection between our work and this theory, as we discussed in the previous response. In particular, we identified the level of self-regulation (measured through the consumption of each species limiting resource) as a key bifurcation parameter in the stability of large microbial communities, which is reminiscent of classic stability analyses [4]. Our first stability condition demonstrates that this classic relationship is not sufficient for stability – that is, the added mechanisms in our model require the effective interaction matrix to satisfy an additional constraint beyond stability.

Lines 242-243. ‘so that each consumer produces an equal mix of all the other resources as it consumes resource i.” From Figure 1 resources other than i are leaked from the consumer after being taken up. Does the term ‘produces’ here mean that the consumers are making resources?

Yes, that is correct. Microbes often leak the resources that they consume as byproducts while they incorporate biomass. In lines 109-132, we describe how we model the two forms of cross-feeding that we consider in more detail.

Lines 235-283. The authors present a number of different consumptions and production structures that satisfy the ‘first criterion’, or the reciprocity of interactions. They test if their analytic stability conditions work on these systems for different parameterizations. This is certainly interesting, but in the absence of presenting empirical evidence that microbial communities follow such reciprocity, there is not

It is true that we do not know whether empirical microbial communities display the structures that have shown are stabilizing. As we have described previously, there are mechanisms that might constrain microbial interaction networks (namely, community assembly and biochemical properties, among others), but we do not yet know whether or not these structures align with our second stability criterion or if they are dominant forces in natural microbial communities. In short, our theoretical results provide new motivation to measure and quantify these interactions in experimental communities, since departures from them might suggest alternative mechanisms that maintain diversity. Moreover, our findings can also guide experimental investigations by suggesting which large-scale properties of the microbial interaction network (ie. the correlation between off-diagonal pairs) may be important for coexistence (as we describe in lines 524-528). More generally, recent work has shown that similar consumer-resource models predict the outcomes of microbial community experiments surprisingly well [3, 5, 6]. Our generalization of these models, therefore, could be similarly useful because it incorporates another known aspect of microbial metabolism into this theoretical framework.

Lines 417-418. “the threshold at which the system becomes unstable is controlled by the magnitude of the imaginary parts of these eigenvalues.” The wording here is strange. I would say that the threshold is of instability may be correlated with the imaginary parts of the eigenvalues, but not ‘controlled’.

We have now edited the language to be “...partly determined by…” in lines 376-377.

Line 453. “Although our model incorporates a more realistic picture of microbial metabolism than previous consumer-resource theory…” Of any previous consumer-resource theory? Is that true?

We did not mean to suggest that our model was the most realistic of all previous consumer-resource theory. We thank the reviewer for pointing out our imprecise language and we have edited this sentence (in lines 427-429) to be:

“Our model incorporates a more mechanistic picture of microbial interactions by considering how essential resources and cross-feeding alter ecological dynamics. At the same time, it is still a highly coarse-grained version of true microbial metabolism.”

Overall, one of our main aims is to clarify how coexistence is maintained in consumer-resource models when (1) both resource competition and exchange are modeled, (2) these processes are tied to a simplified version of internal metabolism and (3) the modeled communities are arbitrarily large. We believe these are the main factors that distinguish our work from previous consumer-resource theory. Our approach is certainly not the only way to model these biological processes. At the time, we show that this approach does refine the conclusions drawn from phenomenological models (lines 443-449).

Line 462-464. “However, allowing non-limiting resources to contribute to consumer biomass greatly complicates our mathematical analysis”. This statement sort of contradicts the ‘more realistic picture’ one above.

We have now modified our model so that consumers incorporate biomass from all resources (including the non-limiting ones), so we have removed this line. As we discussed in the previous response, our model does not incorporate all of the details of microbial metabolism, but it does provide new lessons about how large consumer-resource communities might coexist.

Line 465. “we have also implicitly assumed that the consumer can deplete many of the available resources.” From Eq. (1) is seems they consume all available resources, as there is no washout of resources.

The reviewer is correct in their interpretation of our model. We now lay out this assumption more clearly in lines 104-108.

Line 568-571. “If instead B has eigenvalues with non-zero imaginary part, then the clouds in the spectrum of J will have non-zero width, and some of these eigenvalues will cross the imaginary axis at a small value of n, creating an unstable fixed point.” That is really interesting, but why would B having non-zero imaginary part cause some of the values to cross the imaginary axis? There has to be an underlying reason that can be explained.

The imaginary eigenvalues of B only cause the eigenvalues of the Jacobian to cross the imaginary axis (and hence become unstable) only when the consumer abundance n is small. The imaginary eigenvalues of B create non-zero width in the eigenvalues of

Jacobian, but when n is large, these eigenvalues do not cross the imaginary axis and the system can be stable for relatively small levels of consumption of the limiting resource (see the Unstructured panel in Fig. 3 for an example). However, decreasing the consumer abundance moves the eigenvalues of the Jacobian to the right, towards the imaginary axis, and when these eigenvalues have non-zero width, they will eventually cross the imaginary axis and create an unstable equilibrium (see SI Fig. L). All of that said, we moved this section of the Discussion to the Appendix to increase the clarity of our results (see the section of the Appendix titled “Intuition for why small consumer abundances cause instability”).

I would like to be positive, as the authors are certainly doing ambitious work. But this is a long and (for me) difficult paper; difficult, that is, to keep up with all the assumptions that the authors make, some of which do not seem reasonable, or are at least not justified here. My perseverance was running out about midway through the Discussion of my second reading of the manuscript. There is a lot or repetition. This manuscript reads like an unedited first draft and needs to be streamlined and ways found to get through the complexity. I am not a specialist in randomly assembled webs, but I have considerable experience in reading in this area, and I think this needs to be revised in a very major way; in fact, it needs to be rethought. Also, I don’t know what the criteria are for PLOS Computational Biology papers are, but I don’t see what is new computationally here. As far as I can see, computations are not being used in a novel way. Perhaps the authors could point out what is qualitatively new.

In our revised manuscript, we removed repetitive sentences and unnecessary mathematical details. We also modified our modeling assumptions to make them more biologically realistic. We believe that our work will interest readers of PLOS Computational Biology because it uses numerical analyses, guided by theoretical results, to make predictions about microbial communities which could be tested experimentally. Our theory shows how the processes at the small scale, namely the internal metabolism of different microbial species, can affect the macroecological patterns we observe in nature.

References

[1] Yamagishi JF, Saito N, Kaneko K. Adaptation of metabolite leakiness leads to symbiotic chemical exchange and to a resilient microbial ecosystem. PLOS Computational Biology. 2021;17(6):e1009143. doi:10.1371/journal.pcbi.1009143.

[2] Marsland III R, Cui W, Goldford J, Sanchez A, Korolev K, Mehta P. Available energy fluxes drive a transition in the diversity, stability, and functional structure of microbial communities. PLOS Computational Biology. 2019;15(2):e1006793. doi:10.1371/journal.pcbi.1006793.

[3] Dal Bello M, Lee H, Goyal A, Gore J. Resource–diversity relationships in bacterial communities reflect the network structure of microbial metabolism. Nature Ecology & Evolution. 2021; p. 1–11. doi:10.1038/s41559-021-01535-8.

[4] May RM. Will a Large Complex System be Stable? Nature. 1972;238(5364):413–414. doi:10.1038/238413a0.

[5] Goldford JE, Lu N, Baji´c D, Estrela S, Tikhonov M, Sanchez-Gorostiaga A, et al. Emergent simplicity in microbial community assembly. Science. 2018;361(6401):469–474. doi:10.1126/science.aat1168.

[6] Gowda K, Ping D, Mani M, Kuehn S. Genomic structure predicts metabolite dynamics in microbial communities. Cell. 2022;185(3):530–546.e25. doi:10.1016/j.cell.2021.12.036.
