# Supplementary material for: Stability criteria for the consumption and exchange of essential resources
Source: PLoS Comput Biol. 2022 Sep 8;18(9):e1010521. doi: 10.1371/journal.pcbi.1010521 (PMC9488833; doi:10.1371/journal.pcbi.1010521)
Supplement: Attachment — Submitted filename: ConsVsDepl_ReviewerResponses_2.pdf [file pcbi.1010521.s003.pdf]

In our edited version of the manuscript, we have made a number of substantive changes to address the reviewer comments below. First, we modified Fig. 3 of the manuscript to more faithfully reflect both our simulation data and the accuracy of our predictions. Second, we have more clearly outlined the role of computation in our work and its possible applications. We lay out these connections mainly in our response letter, but also in edits to the Discussion section. Third, we have incorporated the reviewers' suggestions throughout the manuscript, which we believe have further clarified the presentation of our results as well as brought to light interesting connections to previous work.

Note: line numbers refer to the manuscript with changes tracked.

Reviewer #1: I appreciate the authors' efforts in answering my questions. I do not think my main concerns are resolved.

In my perspective, biological significance means the predictions made by the model are closely related to experimentally observed patterns. I do not think the stability criterion in this paper belongs to this category. It depends on all interaction matrix elements, and it is infeasible to measure them in the experiment.

We agree with the reviewer that a critical step in this research area is to test model predictions with controlled experiments, but we believe that this is beyond the scope of the present work. We also agree that it is quite difficult to measure all of the interaction coefficients experimentally. Indeed, one of the main goals for our analysis is to understand how the coexistence of many microbial species depends on large-scale properties of the interaction network, rather than each individual interaction coefficient. Our results suggest that the symmetry of the interaction network has a strong influence on whether or not the community can coexist at small resource supplies. As a result, if future work can identify microbial communities with symmetric interaction networks, for example, by measuring a subset of the interaction coefficients in pairwise experiments or modeling explicit biochemical interactions between microbial species, then our theory suggests that these microbial communities will be stable when resources are scarce. Conversely, if future experiments show that some microbial communities become unstable at small resource supply levels, our theory provides a hypothesis for the structure of the interaction network before any explicit interaction measurements. In short, we have identified a qualitative and robust relationship between community stability and species interactions that can provide insight into a number of future experimental investigations. We describe how our analysis may permit inferences about the interaction coefficients, and how our work might be tested in experiments more generally, in lines 550-574 of the Discussion section.

Second, I agree that the stability criterion may be generalized to the case that the consumer abundances are not identical. But it still requires all resource abundances to be equal, and each species must consume one unique resource(otherwise, the resource abundance is unbound), making the model far from real ecosystems.

The reviewer is correct that our analytical stability criteria require all resource abundances to be equal. To test whether or not this assumption is crucial for our main results, we have now run

additional simulations where the washout rates of the consumers are variable (sampled from a uniform distribution between 0.9 and 1.1), giving rise to variable resource abundances. In a new supplementary figure (copied below), we show that the same type of transition into instability occurs in these new simulations. We also agree with the reviewer that, if consumers do not deplete all the available resources, the resource abundances would diverge in our simulations. In our model, consumers deplete many supplied resources, but consumer growth rates are only sensitive to the abundance of one of these resources. As a result, a single consumer could regulate many resource abundances by depleting them and therefore prevent unbounded resource growth. That said, if there is a resource supplied to the system that no consumers deplete, then its abundance would grow indefinitely. We acknowledge these assumptions about our resource dynamics in lines 105-109.

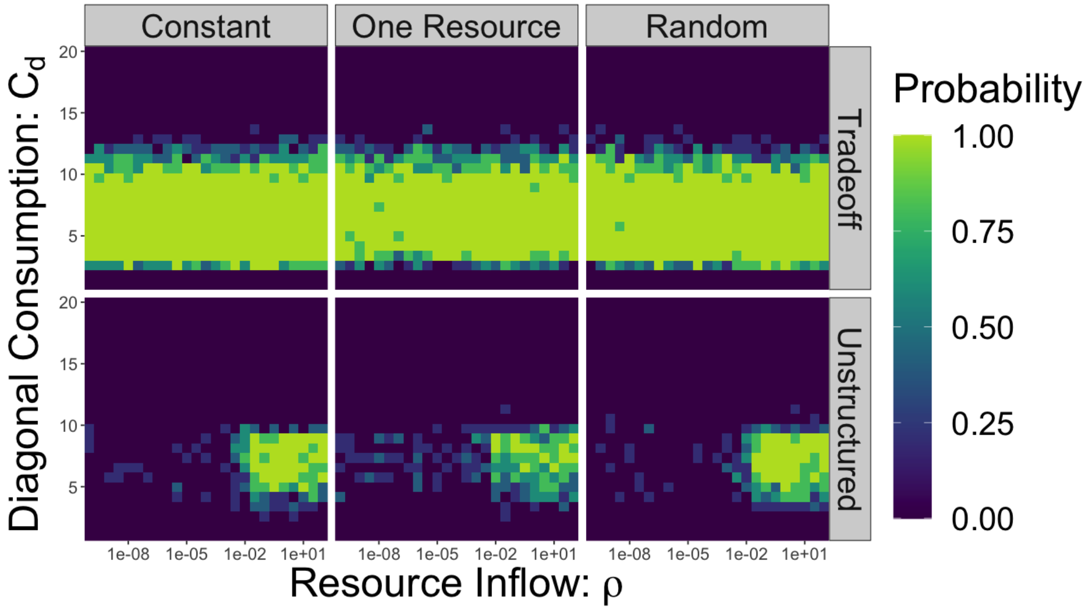

The instability at low consumer abundances is very interesting, and the results are of interest to theoretical ecologists. Unfortunately, I still think it fits more into a theoretical journal than PLOS computational biology.

We are glad that the reviewer finds our main results interesting. Addressing the reviewer's comments have sharpened the manuscript's focus and brought forward our results potential implications for future experiments. We believe that our work is a good fit for PLOS Computational Biology because it connects community-wide stability properties to specific patterns in the interactions between species. This connection may prove helpful in designing computational approaches to inferring microbial interactions. We describe this possible application further in our final response to reviewer #3.

Another comment:

From my understanding, the stability criterion is a sufficient condition for instability. If it is violated, it is wrong. So it is inappropriate to just use the average value in Fig 3, which clearly

shows that it fails for the unstructured case in Appendix Fig C. To avoid confusion, I strongly suggest that the authors add error bars and Figure C in the appendix to Fig 3.

We agree with the reviewer that plotting the mean behavior of our predicted and empirically derived diagonal consumption values does not display the full structure of the data. We have now modified Fig. 3 to more completely capture this set of results (copied below). In panels A-C, we now plot the predicted versus observed values of the diagonal consumption coefficients required for stability in all the replicates of the simulations, across a range of statistics for the consumption coefficients. In panels D-F, we plot histograms of the differences between our predicted and simulated C_d values for the case when the errors are the largest (C_0 = 5 and the CV is 1/sqrt(3)). We have also added a few sentences to the main text to describe these modified plots.

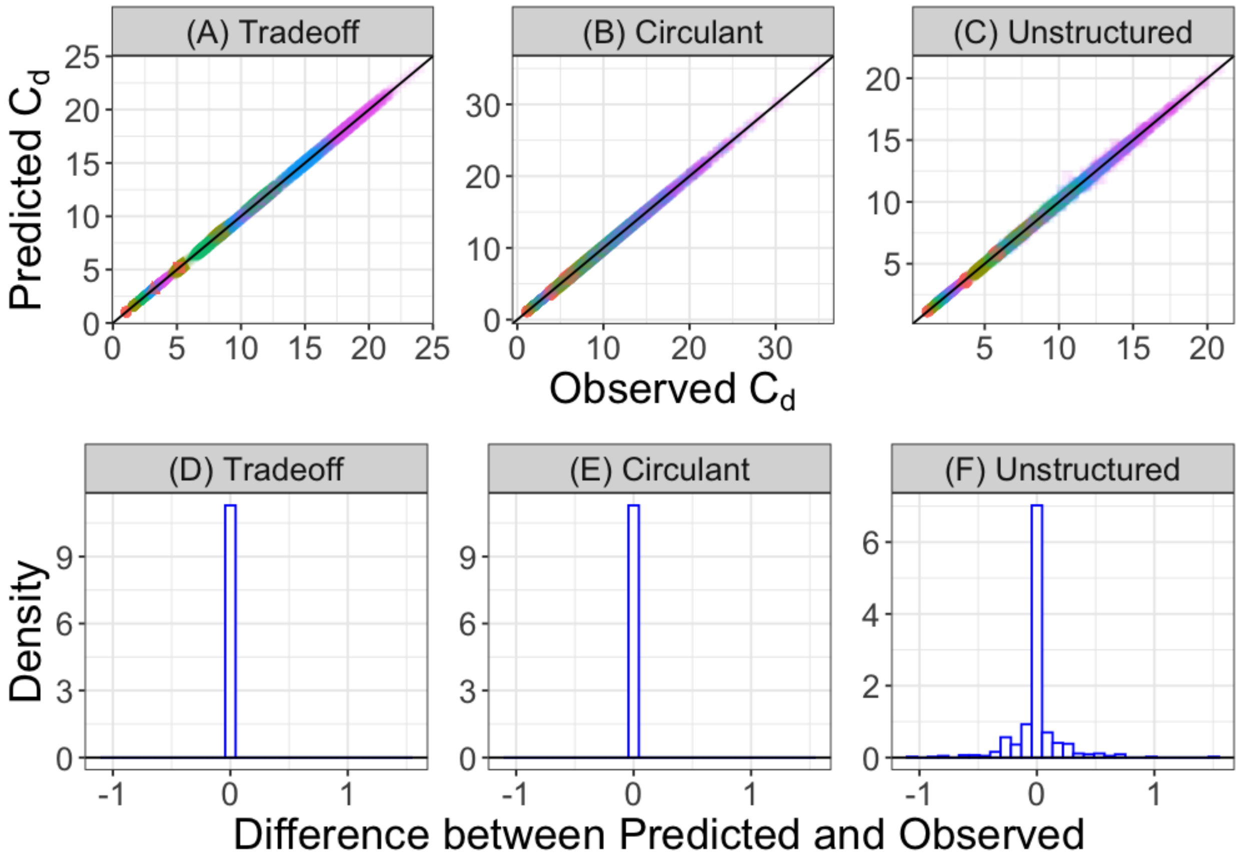

Reviewer #2: Reviewer #2 from the original submission here. The authors have addressed all my comments well, so I am in favor of this manuscript being accepted with no further revision needed. It will make a nice contribution to the burgeoning field of microbial cross-feeding and community stability.

We appreciate the reviewer's positive opinion of the manuscript and believe that addressing the reviewer's comments greatly improved our work.

Reviewer #3: The authors model an arbitrarily large number of microbial species feeding on an equal number of resources. The chemostat model includes cross-feeding, or the use by one organism of metabolites released by other organisms. The authors find conditions for feasibility and stability of the system that depend on structural aspects of the system.

Comments

I reviewed an earlier version of this manuscript. The revised version is much more clearly written and some changes have been made in the model. In particular, all resources contribute to biomass of a consumer, which is more realistic than the original assumption. The changes in the model are explained well in a response to Comment 1 of Reviewer 2, and in the revised text (e.g., Figure 1). This aspect of the model now seems more reasonable. It is also important that it is now stated that the equilibrium is unique and that both criteria for stability (Lines 201-202) are needed for 'stability to be guaranteed' (although it is not clear to me whether that means these are necessary and not just sufficient conditions). It is also important that that stability conditions are shown to hold for interaction networks that are only approximately symmetric. Evolution toward symmetry, that is, toward a more stable system also seems plausible. The authors have also added more support for their assumptions. Overall, this is a greatly improved manuscript.

We are glad that the reviewer finds our manuscript to be much clearer. We also thank the reviewer for the previous comments on our model, which helped us to build the more biologically realistic one that we now focus on. Our stability criteria are sufficient, but not necessary, for stability. We state this in lines 203-204 of the "Sufficient stability criteria for constant abundances" and we now re-emphasize this point in the Results section (lines 292-298).

I have some additional comments, most of which are minor.

Lines 36-49. The paper of Elser et al. (1996) seems relevant and could be cited here or somewhere: Elser, J.J., Dobberfuhl, D.R., MacKay, N.A. and Schampel, J.H., 1996. Organism size, life history, and N: P stoichiometry: toward a unified view of cellular and ecosystem processes. BioScience, 46(9), pp.674-684.

We appreciate the reviewer pointing out this relevant reference and have added a citation to it in the mentioned lines.

Line 59. Change 'which which' to 'which'

Fixed.

Line 91-93. Is this statement correct:. "Under Liebig's law, each consumer's growth rate is determined only by the resource which contributes least to the production of new biomass."? I think it is better stated that, under Liebig's law, the consumer's growth rate is determined by the resource that is available in the environment in the least amount relative to what is needed for the consumer's growth.

We agree with the reviewer that limiting resources under Liebig's law are better interpreted relative to each consumer's requirements rather than as an absolute contribution to each consumer's growth rates. We have changed the language accordingly to: "Under Liebig's law, each consumer's growth rate is determined only by the resource which is least available relative to the consumer's growth requirements."

Lines 111-112. "the excess intake of these resources is cycled back to the environment, possibly following some internal reactions that transform it." Also, in Lines 118-120. "Before being secreted, this internal excess may be converted to other forms through intracellular reactions that do not create new biomass." It is not clear to me whether a single resource is transformed by the 'internal reactions' to a new resource, or whether some combination of the resources that were taken up are combined chemically to a new resource out of more basic elements. The latter sounds much more complicated than what is going on in the equations, but I am not sure. Total biomass is being conserved, but it is not clear that basic building blocks (bio-elements) are being conserved. Lines 437-442 note that this aspect of the modeling will get more attention in the future.

We appreciate the reviewer pointing out this possible source of confusion. In our model, each resource is transformed independently into a single other resource, though more complicated forms of internal metabolism are certainly possible. We have added the following sentence to lines 123-126: "In our model, each resource is transformed independently to produce a set of byproducts, although more complex internal reaction dynamics, where multiple consumed resources react to form byproducts together, are certainly possible [12]." We also added additional clarifying language to the Discussion section (lines 452-463) where we discuss our modeling assumptions and subsequent future directions.

Line 125. It sounds better to change 'a constant fraction' to 'constant fractions'

Fixed.

Line 138. Change 'negative real part' to 'negative real parts'

Fixed.

Line 201. Perhaps more could be said concerning matrix B. I realize that it is discussed in the Supplementary Information (2. Stability Criteria), but maybe more information can be given here. Matrix B comes from Equation (3) when resource diagonal matrix R has equal values r for all elements and matrix C_d is a diagonal matrix. A negative sign is also applied. If symmetric and negative, then all of the eigenvalues of B should be real and negative – or at least non-positive, I believe. So is condition 2 (Line 202) necessary, as the authors state in responses to review comments? I may be missing something.

We have now expanded our discussion of the matrix B with the following sentences immediately after we state our stability criteria: "We derived these conditions through a mathematical analysis of the Jacobian of the equilibrium with constant abundances, which we detail in the "Stability criteria" section of the S1 Appendix. Mathematically, the matrix B is the upper right-hand block of the Jacobian, which measures how the resource abundances change as a

function of the consumer abundances. Interestingly, it is also present in Eq. (3) – a connection we explore further in "Sufficient stability criteria imply feasibility" section of the S1 Appendix." The first condition alone is not sufficient to imply stability because it only ensures that the matrix B is symmetric. B itself can have negative or positive values, depending on the relative sizes of consumption and production of each resource. In addition, a symmetric and entry-wise negative matrix can still have eigenvalues with positive real parts, and hence create an unstable equilibrium. As a result, we need the second condition to guarantee stability.

Line 242. It is perhaps not necessary to say "In the main text"

Fixed.

Lines 309-310. "low consumer abundances lead to instability". Low consumer abundances result from small values of input, ro, which usually relates to the high residence time of resources in the system. It is known, at least for simple model systems, that the shorter the residence time is, the more resilient the system is; e.g., DeAngelis, D.L., 1980. Energy flow, nutrient cycling, and ecosystem resilience. Ecology, 61(4), pp.764-771.. The effect of high throughflow (low residence time) could make unstructured systems here less vulnerable to the occurrence of local instability by pushing real parts of eigenvalues to larger negative values.

We thank the reviewer for pointing out this interesting connection. We now cite this reference and include two sentences on this possible interpretation of our results in the Discussion section, which we copy here: "The resilience of a food web (defined as the speed at which the abundances return to equilibrium after a perturbation) has been shown to increase as the residence time of nutrients in the system decreases [54]. One possible interpretation of our results is that, when resource supply is low, resources remain in the community for longer times, inducing instability, but further work is needed to understand this connection more completely."

Lines 369-376. It is shown that the imaginary parts of the eigenvalues of B to the minimum resource inflow level at which the community can exist. Is this simply intuitive; i.e., as fluctuations would more seriously affect stability (possibly cause population values to become negative) if average population abundances are small due to low resource inflow rates?

We thank the reviewer for raising this important point. In the simulations of the dynamics of our model (Fig. 1(C) and 6), we scale the size of the perturbations we add to the original equilibrium abundances by the size of these equilibrium abundances themselves. Therefore, the resulting behavior is not due to the relative sizes of the equilibrium abundances and the fluctuations around them. Moreover, the fact that equilibria with low consumer abundances give rise to Jacobians with eigenvalues that have positive real parts means that fluctuations of *any size* will cause the dynamics to diverge from equilibrium. As a result, the instability from these low consumer abundances is not the same as the loss of coexistence that results from small population sizes and demographic stochasticity. Indeed, we observe the same transition into instability in our model when the consumer abundances are not small in absolute value (in SI Fig. F they are order 1), but instead are small relative to an expression of the other parameters in the model. As an example, Eq. S15 relates the size of the consumer abundances required for stability in terms of the other model parameters for the no-cross-feeding case. In short, the

consumer abundances need only be small relative to a threshold that emerges from our mathematical analysis. Our results are not driven by small consumer abundances alone.

Line 375. Change ‘imaginary part’ to ‘imaginary parts’

Fixed.

Line 481. Change ‘they require’ to ‘it requires’

Fixed.

This is a vastly improved manuscript. The methods and results are clear and the limitations of the modeling are discussed. The authors have cited appropriate references and put their work into context. The four main results seem to be sound and interesting, especially that symmetric interaction structures are protected from instability at low resource flow. My final comment is perhaps the authors should emphasize more why this manuscript is aimed at PLOS Computational Biology. Certainly, computations are important in the simulations, but it is not clear why the manuscript is more appropriate for PLOS Computational Biology than say, any number of theoretical biology journals.

We are very glad that the reviewer believes the changes to our manuscript have improved it. We agree with the reviewer that, in our work, we have used extensive, mathematically-guided simulations to test whether or not our analytical stability criteria apply beyond the regime in which we proved them. Besides this numerical approach, we believe our work is appropriate for PLOS Computational Biology because it shows how specific patterns in the vast space of possible microbial interactions give rise to predictable outcomes. Previous work modeling microbial communities has attempted to infer all of the species interactions from microbial abundance data [1,2]. In this inference approach, it is necessary to impose constraints on the interaction parameters, because the space of all possible interactions is so large. More recent computational work has tied this inference procedure to well-studied consumer-resource models [3,4]. An interesting possible application of our theory, therefore, is to use the relationships we have derived between interaction networks and coexistence outcomes to further constrain this inference problem. In this way, our work may facilitate future computational approaches to the modeling of microbiome communities. We have added a brief discussion of this possible application to the Discussion in lines 559-563.

References

[1] Stein, Richard R., et al. "Ecological modeling from time-series inference: insight into dynamics and stability of intestinal microbiota." PLoS computational biology 9.12 (2013): e1003388.

[2] Bucci, V., Tzen, B., Li, N., Simmons, M., Tanoue, T., Bogart, E., ... & Gerber, G. K. (2016). MDSINE: Microbial Dynamical Systems INference Engine for microbiome time-series analyses. Genome biology, 17(1), 1-17.

[3] Wang, T., Goyal, A., Dubinkina, V., & Maslov, S. (2019). Evidence for a multi-level trophic organization of the human gut microbiome. PLoS computational biology, 15(12), e1007524.

[4] Goyal, A., Wang, T., Dubinkina, V., & Maslov, S. (2021). Ecology-guided prediction of cross-feeding interactions in the human gut microbiome. Nature communications, 12(1), 1-10.
